# Development and validation of a food photography atlas for portion size estimation of traditional Croatian dishes in adults

**DOI:** 10.3389/fnut.2026.1956906

**Published:** 2026-10-07

**Authors:** Ivana Rumbak, Ana Ilić, Tea Knezović, Ana Jurčić, Martina Pavlić, Lidija Šoher, Daniela Čačić Kenjerić, Jasna Pucarin-Cvetković, Darja Sokolić

**Affiliations:** 1 Laboratory for Nutrition Science, Department of Nutrition, Food Quality and Safety, University of Zagreb Faculty of Food Technology and Biotechnology, Zagreb, Croatia; 2 Centre for Food Safety, Croatian Agency for Agriculture and Food, Osijek, Croatia; 3 Sub-Department of Nutrition, Department of Food and Nutrition Research, Faculty of Food Technology Osijek, Josip Juraj Strossmayer University of Osijek, Osijek, Croatia; 4 Department of Environmental and Occupational Health and Sports Medicine, Andrija Štampar School of Public Health, School of Medicine, University of Zagreb, Zagreb, Croatia; 5 Division for Environmental Health, Croatian Institute of Public Health, Zagreb, Croatia

**Keywords:** dietary assessment, food atlas, food consumption survey, food intake estimation, food photography, portion size, traditional food, validation study

## Abstract

**Background:**

Accurate portion size estimation is an important component of dietary assessment. Food photography atlases, which are among the most commonly used portion size measurement aids, are designed to take into account the cultural context and dietary patterns of the target population. Therefore, the aim of this study was to develop and validate food photography atlas of traditional Croatian dishes for adults.

**Methods:**

The 30 dishes were selected, in accordance with the gastronomic regions defined by Croatian National Tourist Board. The portion sizes were determined using data from the first Croatian national food consumption survey of the adult population, conducted in accordance with the EU Menu methodology. The photographs were validated by comparing them with the actual dishes served to 110 participants over a three-day period. Validity assessment included mean differences between actual and estimated portion sizes using the Wilcoxon signed-rank test, correlations between actual and estimated portion sizes, Bland– Altman limits of agreement, and quadratic weighted Cohen’s kappa.

**Results:**

Of the 30 selected traditional dishes, no significant differences between actual and estimated portion sizes were observed for 46.7% of dishes, whereas portion sizes were underestimated for 30% and overestimated for 23.3%. For half of the dishes, at least 50% of the estimates were within ±10% of the differences between actual and estimated portion size. Spearman’s correlation showed a strong association between actual and estimated portion size for all 30 dishes. Bland–Altman analysis demonstrated good agreement between actual and estimated portion sizes, with low systematic bias and narrow limits of agreement for single-unit and countable dishes. In 66.7% of dishes, more than half of the estimations were correct, while across all 30 dishes the proportion of distant estimations ranged from 0.0 to 31.0%. Quadratic weighted Cohen’s kappa indicated almost perfect agreement for 29 dishes and substantial agreement for one dish.

**Conclusion:**

These results support the validity of the food photography atlas for estimating portion sizes of traditional dishes among adults in Croatia.

## 1 Introduction

Collecting accurate data on food intake is of utmost importance in epidemiological studies for determining the impact of nutrition on health outcomes (1). Adequate assessment of food consumption is also crucial in nutritional counseling for identifying potential nutritional deficits and monitoring the success of counseling (2). A weighed food diary is considered a detailed dietary assessment method, however, when this is not feasible, dietary intake can be assessed using 24-h recalls, in which kitchen utensils and descriptive portion sizes (e.g., 1 egg, 1 slice of bread) can be used for portion estimation. Furthermore, it is common to use portion size estimation aids as visual aids to improve the accuracy of portion size estimation and reduce participant burden (3, 4). To achieve the highest possible accuracy when estimating food intake, food photographs should be designed according to several recommendations (5). Food photographs should cover a representative range of portion sizes consumed by the population for which the food photography atlas is intended. Also, photographs should include commonly consumed dishes and reflect the cultural context of the population. Despite recommendations for accurately calculating portion sizes and photographing food, estimates based on food photographs may still contain errors in measured food consumption, ranging from 4 to 50% (6–14). Therefore, it is necessary to determine the potential magnitude and direction of error when estimating portion sizes from food photographs (15).

Croatian gastronomy has developed under the influence of many cultures that have prevailed in specific regions of Croatia throughout history. The influence of the Roman Empire, Greek culture, and the Venetian Republic is evident in the coastal regions, and Austro-Hungarian and Turkish influences are characteristic of the continental regions of Croatia (16, 17). Moreover, Croatia is geographically very diverse, with different crops and livestock raised in each region, which also affects the eating habits of the population. Therefore, the Croatian National Tourist Board divides Croatian gastronomy into several gastronomic regions (Dalmatia, Istria and Kvarner, Slavonia, Lika, Central and North Croatia) (16). The Croatian version of the Mediterranean diet is listed as Intangible Cultural Heritage of Humanity (18), while the European Commission’s register of geographical indications, eAmbrosia, lists more than 70 Croatian products (19). The uniqueness of Croatian gastronomy lies in the preservation of traditional recipes and their use during special occasions, as well as in everyday life. Despite gradual changes in lifestyle and eating habits among Croatian inhabitants, the custom of consuming cooked meals in several courses still prevails, especially on weekends, when the sociocultural aspect of dining is also emphasized (17).

The diversity of traditional regional dishes presents a challenge for estimating portion sizes and highlights the need to develop tools adapted to the Croatian dietary context. Although a food photography resource has been developed for Croatian children (20), no corresponding validated resource for Croatian adults is currently available. This shortcoming may represent a limitation in nutrition research, but also in nutritional practice, where accurate assessment of food intake is of key importance. Since food photography atlases should reflect foods and dishes that are recognizable to the target population, using atlases developed for other countries may limit the accuracy of assessing food intake in the Croatian population. Therefore, the aim of this study was to develop a food photography atlas for adults featuring traditional Croatian dishes and to assess its validity in estimating portion sizes.

## 2 Materials and methods

The present study was conducted in three phases: dish selection and portion size estimation, development of a food photography atlas, and validity assessment. The principles of the Declaration of Helsinki were observed throughout the study, and ethical approval for its implementation was obtained from the Committee for Ethical Issues of the University of Zagreb, Faculty of Food Technology and Biotechnology (Class: 004-01/24-01/01; Reg. No. 251-69-01-24-4).

### 2.1 Dish selection and portion size estimation

The dishes were selected using information from the Croatian National Tourist Board, which identifies traditional Croatian dishes for each gastronomic region (16). A total of 30 dishes were chosen, 20 of which are from specific gastronomic regions (Dalmatia: 4 dishes; Istria and Kvarner: 3 dishes; Slavonia: 4 dishes; Lika: 2 dishes; Central and North Croatia: 7 dishes), 5 are traditional Croatian desserts, and 5 additional dishes were included because they are frequently consumed in Croatian regions and originally come from Eastern European and Balkan cuisines.

Portion size estimation was carried on according to the EFSA recommendations from the Pilot study for Assessment of Nutrient intake and food Consumption Among Kids in Europe (PANCAKE) (5). For each dish, portion size was estimated from three nonconsecutive 24-h food recalls completed by 2002 adults (51.5% men; 18–64 years) enrolled in a Croatian national food consumption survey on adult population (NIPNOP-HAH-2011-2012) (21, 22). Details related to the methodology for collecting 24-h recalls and their processing are described in detail in the report (21). For each dish, four portion sizes were estimated using an equidistant approach. The smallest portion corresponds to the 5th percentile and the largest to the 95th percentile, with the 35th and 65th percentiles representing the intervals between these portion sizes. However, during food preparation and weighing before photography, minor weight adjustments were made to six food items (grilled fish with Swiss chard—fish only; roasted lamb with potatoes—lamb only; stuffed cabbage rolls *“Sarma”* with mashed potatoes—stuffed cabbage rolls *“Sarma”* only; kebabs *“Ćevapi”* in flatbread *“Lepinja”*—both kebabs *“Ćevapi”* and flatbread *“Lepinja”*; baked cheese pastry *“Štrukli”*). This weight adjustment was based on raw calculated percentile weights that produced uncharacteristic, nonstandard visual presentations (e.g., one and two-thirds of a fish). By adjusting these weights, we ensured that the photographed dishes reflect typical serving practices in households and other settings, facilitating more accurate visual estimation. The final list of dishes with corresponding portion sizes is provided in Supplementary Table S1.

### 2.2 Development of a food photography atlas

Food photographing was conducted over 4 days, following EFSA recommendations (5). The dishes were ordered from restaurants or prepared just before photographing, while desserts and foods such as cheese and cold cuts were purchased from stores. The dishes were weighed on a digital scale (Daewoo Electronic Kitchen Scale, DKS-2055) and served (Figure 1) on white plates (large deep plate: inner diameter 15.5 cm, outer diameter 22.5 cm; large shallow plate: inner diameter 18 cm, outer diameter 22.5 cm) and in a transparent bowl (inner diameter 8.0 cm, outer diameter 16.5 cm). Dishes consisting of several food items were served with side dishes on the left and meat or fish on the right. Other dishes were served in the center of the plate or in bowls. The dishes were photographed on a blue background, with cutlery placed next to the plate or bowl. The camera used for the photography was a Canon EOS 5D Mark III. Desserts and similar foods were photographed at an angle of 35°, dumplings stuffed with plum, baked cheese pastry *“Štrukli”* and pancakes at 45°, and other cooked food at 52°. Figure 2 shows example photographs of traditional Croatian dishes.

**Figure 1:**
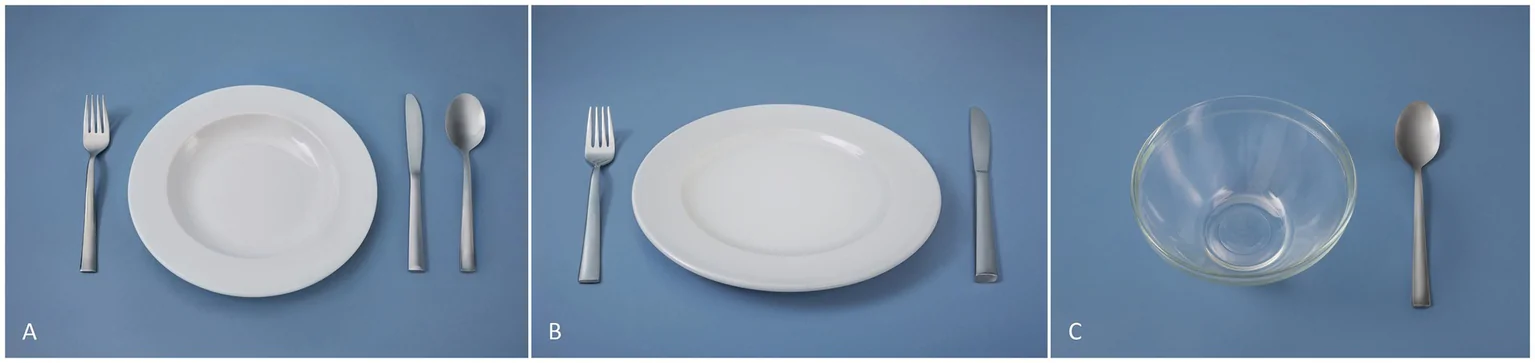
Plates and bowls used for serving food and background for food photography. **(A)** Large deep plate; **(B)** large shallow plate; **(C)** transparent bowl.

**Figure 2:**
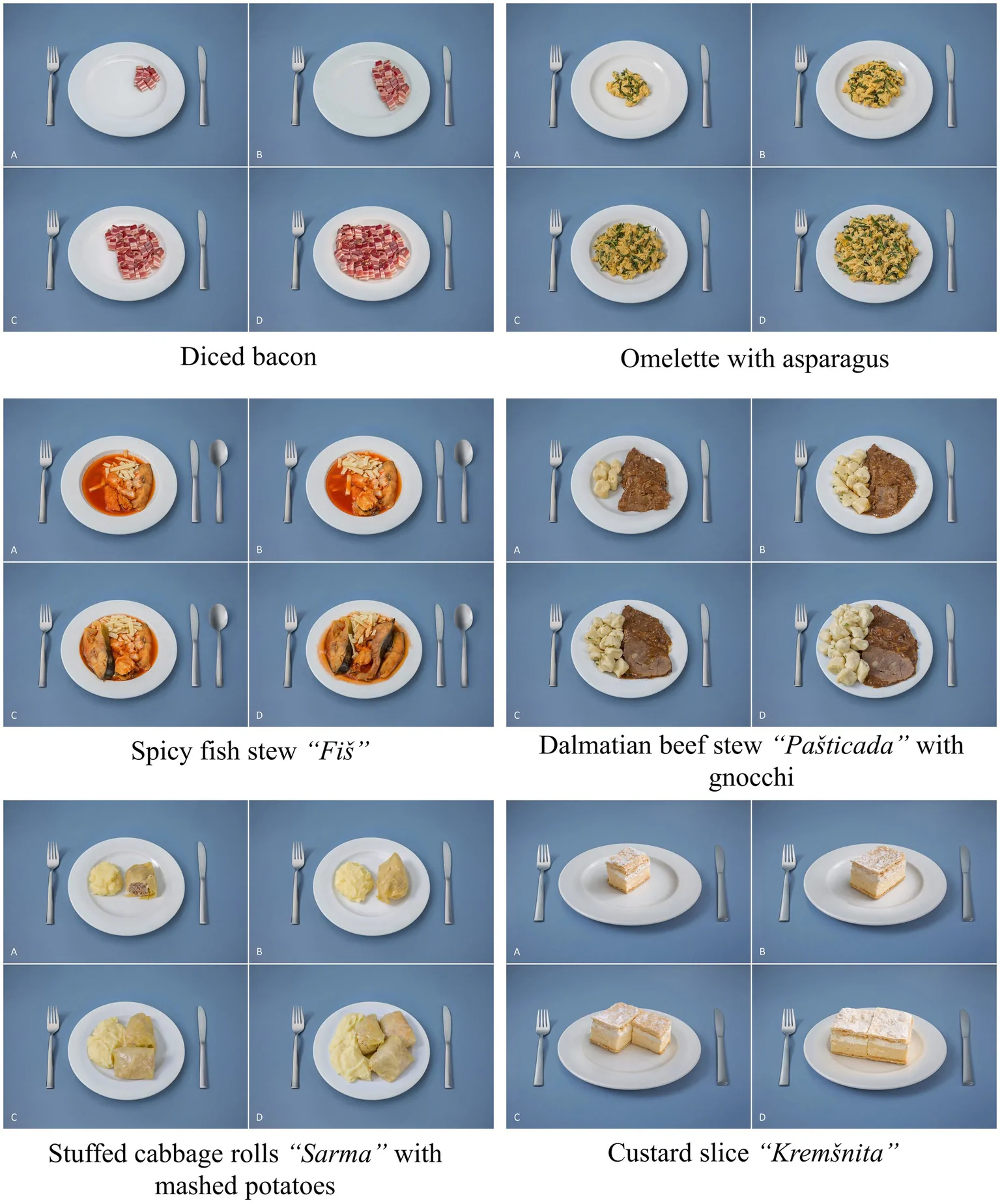
Examples of photographs of traditional Croatian dishes. **(A)** 5th percentile; **(B)** 35th percentile; **(C)** 65th percentile; **(D)** 95th percentile.

### 2.3 Validity assessment

The third phase, during which the photographs were validated, was conducted over 3 days in the Laboratory for Nutrition Science at the University of Zagreb Faculty of Food and Biotechnology. The study included in total of 110 participants (76 on the first day, 87 on the second day, and 84 on the third day). Based on results from existing studies on food photograph validation (7–10, 14, 23), it was calculated that a minimum of 63 estimates/participants are required to validate each dish (to detect 25% differences; power at 80%, *α* = 0.05) (15).

Each day, sets of photographs of 10 dishes were validated and grouped to optimize food preparation and serving logistics (e.g., delivered from the restaurant, prepared on site before validation, or purchased from the store). For each set of photographs, ±1/3 of the difference between a picture and the adjacent picture was calculated. In total, 12 different portion sizes were served for each dish. Estimation was structured across 12 estimation stations (Figure 3), with each station displaying one portion size per dish. To eliminate order bias, both the portion size assigned to each estimation station and the sequential arrangement of dishes within each station were randomized. The dishes were weighed on the same digital scale and served on the same white plates and transparent bowls used during the photography development phase.

**Figure 3:**
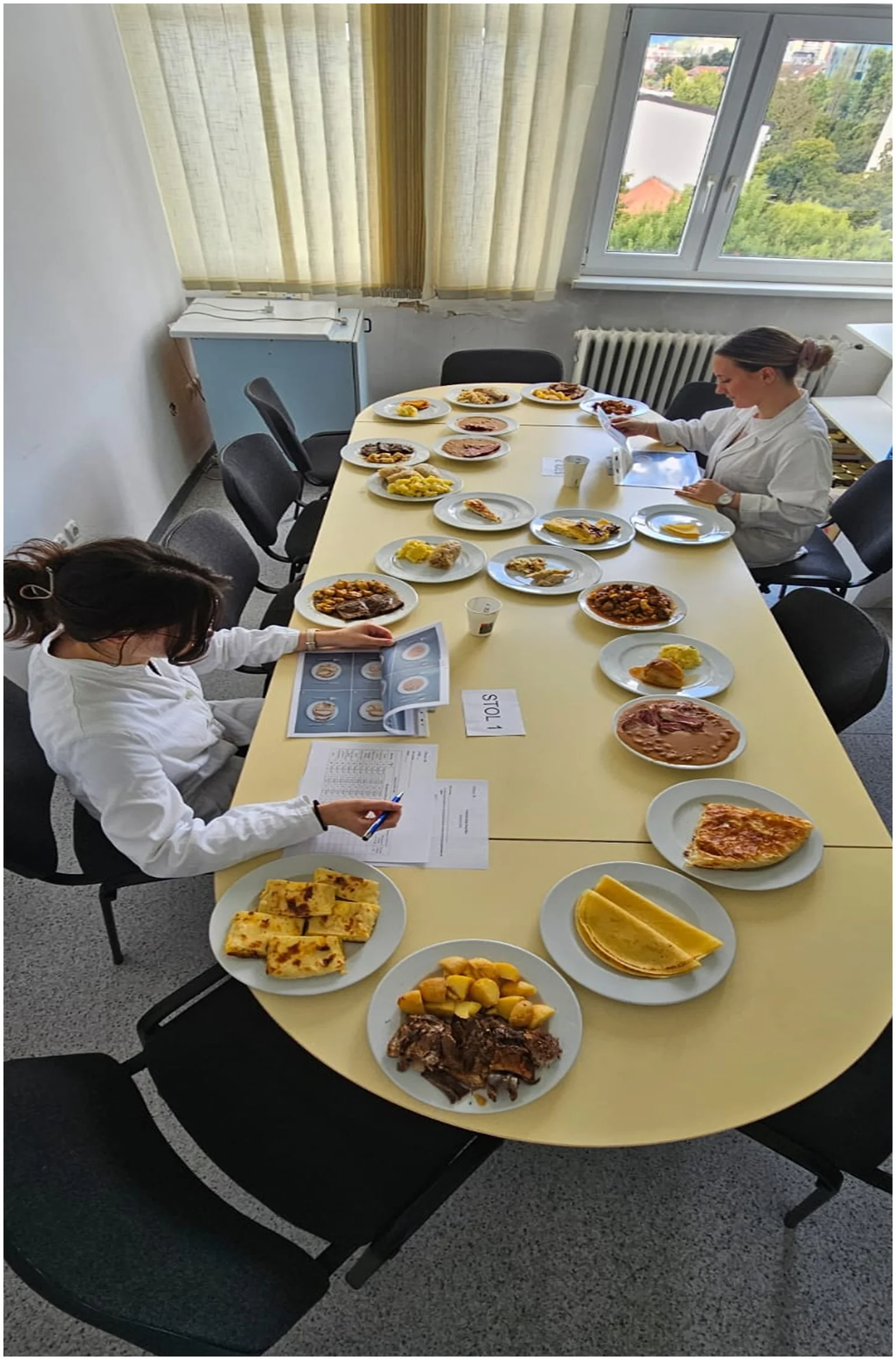
Example of the estimation stations with served dishes and questionnaires.

Upon arrival, the participants were informed about the study protocol and signed an informed consent form to participate. Afterward, they completed a general questionnaire with personal data (date of birth, sex, body weight, height, level of education, and profession). Participants then proceeded to their assigned estimation station. At each estimation station, they were presented with four reference photographs and an evaluation questionnaire. To account for the ±1/3 difference between a photograph and the adjacent portion sizes, the questionnaire allowed selection from nine estimation codes (−A, A, A/B, B, B/C, C, C/D, D, and D+). After completing the portion size estimation, participants filled out a questionnaire on the perceived difficulty of the portion size estimation.

### 2.4 Statistical analysis

Descriptive analysis, assessment of difference and agreement between actual and estimated portion sizes were performed using IBM SPSS Statistics (version 23.0; IBM Corp., Armonk, NY, USA) and Microsoft Excel 2016 (version 16.0.5413.1000; Microsoft Corporation, Redmond, WA, USA). In all tests, *p* < 0.05 was considered significant.

Categorical data were presented as percentages, and continuous data were presented as medians (interquartile ranges) because their distributions were skewed, as assessed by the Shapiro–Wilk test. For each dish, the medians of the actual and estimated portion sizes were presented, and their difference (difference (*g*) = estimated size − portion size) and relative difference (relative difference (%) = ((estimated size − portion size)/portion size) × 100) were calculated. Additionally, the percentages of participants with ±10, ±25, ±50, and ±75% differences between actual and portion size were observed. The Wilcoxon signed-rank test was used to assess overestimation and underestimation between actual and estimated portion sizes. To control for multiple comparisons across the 30 dish evaluations, *p*-values from the Wilcoxon signed-rank tests were adjusted using the Benjamini–Hochberg false discovery rate procedure. Spearman’s rank correlation coefficient was used to assess the linear relationship between actual and estimated portion sizes. For visual assessment of agreement between actual and estimated portion sizes, Bland–Altman plot analysis was performed. In the plots, the differences between actual and estimated portion sizes were plotted against the mean of the actual and estimated portion sizes. Non-parametric limits of agreement (LOA) were set at the 2.5th and 97.5th percentiles. Agreement was evaluated by considering the practical acceptability of the LOA width in relation to the range of portion sizes represented in the food atlas, along with the distribution of estimation errors across portion sizes (24, 25). To assess inter-rater agreement between actual and estimated portion size, quadratic weighted Cohen’s kappa (*κ_w_*) was used, which accounts for agreement expected by chance while allowing partial agreement between adjacent categories (26). For the purpose of this assessment, estimated portion sizes were converted to an ordinal 1–9 scale (−A = 1, A = 2, A/B = 3, B = 4, B/C = 5, C = 6, C/D = 7, D = 8, and D+ = 9). Additionally, 12 actual portion sizes were assigned to the same ordinal 1–9 scale using cutoff boundaries defined at ±1/3 of the weight difference between adjacent photographed portion sizes. Using these ordinal categories, estimates were classified into three levels of accuracy: correct portion sizes, adjacent estimates (difference of ±1), and distant estimates (difference of >±1). Cohen’s *κ_w_* values of 0.6–0.8 was interpreted as substantial agreement, while values >0.8 were interpreted as almost perfect agreement.

Acceptable agreement was evaluated using primary and secondary criteria. The primary criterion was overall agreement, defined as Cohen’s kappa (*κ_w_* > 0.6) and Spearman’s rank correlation (*r* > 0.8). The secondary criterion required that more than 50% of estimates fell within ±25% of the actual portion size. Systematic over- and underestimation was defined as a relative difference of more than 20% between actual and estimated portion sizes. Finally, Bland–Altman plots visually assessed systematic bias and overall practical acceptability of errors across portion sizes.

## 3 Results

The validation phase of the food photography atlas for portion size estimation of traditional Croatian foods in adults enrolled 110 participants (Table 1), with a median age of 35.9 years (30.6–44.6). Most participants were women (65.6%), had a faculty or doctoral degree (70.9%), and worked in professions not related to food (75.5%). In addition, most had not previously participated in any validation study of a food photography atlas (71.6%) or used one to estimate portion size (78.9%). The study lasted 3 days, and 49.1% of participants took part on all 3 days. Each day, the study sample included enough participants to validate the food photography atlas. Regarding weight status, 50.0% of participants had a normal BMI, while 48.2% were overweight or had obesity.

**Table 1:** Characteristics of the participants in the validation phase of the study.

Characteristics	Total sample (*N* = 110)
Age (year)	35.9 (30.6–44.6)
Sex (%)	
Women	65.6
Men	34.4
Education level (%)	
Primary education	0.9
Secondary education	15.5
Professional bachelor’s degree	12.7
Faculty (bachelor’s and master’s degree)	36.4
Doctorate degree	34.5
Profession (%)	
Food related^1^	24.5
Non-food related	75.5
Previous participation in food atlas study (%)	
No	78.9
Yes	21.1
Previous use of food atlas for portion size estimation (%)	
No	71.6
Yes	28.4
Body weight (kg)	75.0 (61.0–88.0)
Body height (cm)	172.0 (166.0–178.0)
Body mass index (kg/m^2^)	24.6 (21.3–27.7)
Body mass index category (%)	
Underweight	1.8
Adequate	50.0
Overweight	31.8
Obesity	16.4
Study attendance (%)	
One day	24.5
Two days	26.4
Three days	49.1

Data are presented as median (interquartile range) or percentages. ^1^Included nutritionists, students of Nutrition study, chefs and butcher.

Table 2 presents the median actual portion size and the median estimated portion size, along with their difference and relative differences. Of the 30 dishes, the Wilcoxon signed-rank test did not find a significant difference between actual and estimated portion sizes for 14 of them. However, after adjusting for multiple comparisons using the Benjamini–Hochberg false discovery rate procedure, the differences for kebabs *“Ćevapi,”* dumplings stuffed with plum, and custard slice *“Kremšnita”* were no longer significant. This meant that for 17 of the 30 dishes, no statistically significant difference was found between the actual portion size and the estimated portion size. The significant underestimation was found for sliced (adjusted *p* = 0.003) and diced (adjusted *p* = 0.046) bacon, omelette with asparagus (adjusted *p* = 0.003), brown roux soup *“Prežgana juha”* (adjusted *p* = 0.003), risotto with squid (adjusted *p* = 0.003), octopus and potato cooked under the bell (adjusted *p* = 0.003), spicy fish stew *“Fiš”* (adjusted *p* = 0.003) and Slavonian meat stew *“Čobanac”* (adjusted *p* = 0.003). Significant overestimation was found for cottage cheese with sour cream (adjusted *p* = 0.003), bean stew with smoked meat (adjusted *p* = 0.018), stuffed cabbage rolls *“Sarma”* with mashed potatoes (adjusted *p* = 0.003), kebabs *“Ćevapi”* in flatbread *“Lepinja”* (adjusted *p* = 0.003), and walnut roll (adjusted *p* = 0.003). Of 16 dishes, the median relative differences were less than 5% for 12 dishes, between 5 and 10% for 1 dish, and greater than 10% for 3 dishes. Moreover, for 28 dishes, 60% or more of the estimates were within ±25% of the relative difference. According to the Spearman correlation coefficient (*p* < 0.001 for all), the estimated portion size of all 28 dishes was very strongly correlated with the actual portion size, except for Dalmatian beef stew *“Pašticada”* with gnocchi (*r* = 0.796) and Slavonian meat stew *“Čobanac”* (*r* = 0.740), for which the correlations were strong.

**Table 2:** Median values and differences between the actual portion size and the estimated portion size of dishes from food photography atlas of Croatian traditional foods.

Dishes	Observations	Actual portion size	Estimated portion size	Difference	Wilcoxon signed-rank	FDR adjustment	Spearman correlation	Relative difference	Percentage of participants within relative difference			
	(*n*)	(*g*)	(*g*)	(*g*)	*p* value	adjusted *p* value	(*r*)	(%)	±10%	±25%	±50%	±75%
Cheese “*Škripavac”*	76	101.3 (39.3–196.3)	129.0 (46.0–230.0)	0.0 (0.0–18.7)	0.193	0.252	0.969^**^	0.0 (0.0–18.7)	30.3	68.4	92.1	96.1
Dry-cured ham Prosciutto	76	60.0 (32.5–140.0)	63.8 (37.5–123.8)	0.0 (−7.5 to 5.0)	0.425	0.490	0.945^**^	0.0 (−11.6 to 15.4)	39.5	77.6	90.8	98.7
Dry spice cured sausage “*Kulen”*	76	102.0 (45.0–169.0)	126.0 (54.0–158.5)	2.3 (0.0–24.0)	0.060	0.100	0.929^**^	3.7 (0.0–25.8)	34.2	64.2	85.5	97.4
Bacon (diced)	76	98.0 (47.3–162.0)	89.0 (62.0–150.5)	0.0 (−11.5 to 0.0)	0.020	0.046	0.974^**^	0.0 (−9.2 to 0.0)	48.7	80.3	97.4	100.0
Bacon (sliced)	76	81.3 (30.7–160.7)	64.0 (39.0–116.0)	−8.3 (−26.0 to 0.0)	<0.001	0.003	0.959^**^	−8.8 (−25.0 to 0.0)	38.2	67.1	94.7	100.0
Omelette with asparagus	84	222.3 (96.3–378.3)	210.0 (58.0–247.0)	−26.1 (−65.7 to 0.0)	<0.001	0.003	0.966^**^	−14.3 (−33.2 to 0.0)	34.5	70.2	100.0	–
Baked cheese pastry *“Štrukli”*	87	227.0 (125.3–346.7)	184.0 (140.0–385.0)	0.0 (−19.2 to 29.3)	0.230	0.288	0.957^**^	0.0 (−9.5 to 11.0)	58.6	82.8	98.9	100.0
Cottage cheese with sour cream	76	130.0 (82.7–236.3)	159.5 (107.0–266.0)	12.2 (0.0–59.3)	<0.001	0.003	0.935^**^	13.9 (0.0–50.2)	35.5	53.9	73.7	90.8
Phyllo pastry with cheese *“Burek”*	87	236.5 (117.0–532.3)	285.8 (106.5–631.0)	0.0 (−21.0–10.5)	0.250	0.300	0.977^**^	0.0 (−9.3 to 10.9)	44.8	83.9	98.9	100.0
Brown roux soup *“Prežgana juha”*	76	217.5 (170.0–287.5)	195.0 (176.3–262.5)	−3.1 (−25.0 to 0.0)	<0.001	0.003	0.918^**^	−1.7 (−13.7 to 0.0)	61.8	90.8	98.7	100.0
Grilled fish with Swiss chard	84	624.8 (293.5–908.0)	624.8 (331.8–960.5)	0.0 (−52.5 to 38.3)	0.138	0.188	0.983^**^	0.0 (−8.7 to 5.8)	66.7	91.7	97.6	100.0
Fried small fish	84	295.5 (131.3–415.7)	295.5 (79.0–446.0)	0.0 (−39.7 to 39.7)	0.680	0.730	0.929^**^	0.0 (−16.9 to 20.0)	35.7	59.5	95.2	100.0
Risotto with squid	84	367.3 (240.0–455.3)	290.0 (215.0–480.0)	0.0 (−38.7 to 0.0)	<0.001	0.003	0.930^**^	0.0 (−10.8 to 0.0)	50.0	86.9	97.6	100.0
Octopus and potato cooked under the bell	84	326.0 (155.7–466.7)	237.0 (176.0–326.0)	−59.3 (−178.0 to 0.0)	<0.001	0.003	0.946^**^	−23.2 (−33.4 to 0.0)	17.9	48.8	97.6	100.0
Spicy fish stew *“Fiš”*	84	330.0 (250.0–400.0)	315.0 (240.0–360.0)	−17.5 (−60.0 to 0.0)	<0.001	0.003	0.847^**^	−4.5 (−18.2 to 0.0)	57.1	84.5	96.4	98.8
Bean stew with smoked meat	87	373.8 (216.7–556.0)	395.8 (245.0–603.3)	11.0 (0.0–56.7)	0.007	0.018	0.934^**^	2.9 (0.0–14.5)	47.1	80.5	88.5	97.7
Roasted turkey with pasta *“Mlinci”*	87	399.4 (205.3–563.4)	438.0 (234.5–626.0)	0.0 (−19.4 to 87.5)	0.074	0.111	0.938^**^	0.0 (−4.9 to 22.1)	47.1	70.1	94.3	98.9
Dalmatian beef stew *“Pašticada”* with gnocchi	87	481.3 (324.7–723.4)	512.0 (420.0–670.5)	0.0 (−92.3 to 52.9)	0.506	0.562	0.796^**^	0.0 (−17.1 to 11.0)	42.5	65.5	95.4	98.9
Slavonian meat stew *“Čobanac”*	87	371.0 (292.0–425.0)	323.0 (230.0–440.0)	−15.3 (−66.0 to 0.0)	0.001	0.003	0.740^**^	−4.3 (−20.3 to 0.0)	42.5	74.7	95.4	98.9
Cooked vegetables *“Sataraš”* with roasted meat	84	429.0 (263.7–575.0)	487.0 (313.0–619.0)	0.0 (0.0–44.0)	0.081	0.116	0.957^**^	0.0 (0.0–11.5)	60.7	90.5	96.4	97.6
Roasted lamb with potatoes	87	457.4 (242.0–592.0)	471.8 (267.0–630.5)	0.0 (−25.0 to 50.0)	0.072	0.111	0.947^**^	0.0 (−5.8 to 11.5)	56.3	82.8	96.6	100.0
Stuffed pepper with mashed potatoes	87	528.4 (339.3–842.7)	598.0 (389.0–781.5)	0.0 (−24.8 to 69.6)	0.586	0.628	0.942^**^	0.0 (−6.6 to 14.5)	58.6	75.9	98.9	–
Stuffed cabbage rolls *“Sarma”* with mashed potatoes	87	445.0 (285.7–698.7)	510.0 (315.0–793.0)	0.0 (0.0–65.0)	<0.001	0.003	0.937^**^	0.0 (0.0–14.6)	52.9	71.3	90.8	98.9
Kebabs *“Ćevapi”* in flatbread *“Lepinja”*	84	306.5 (174.7–508.3)	482.5 (158.0–560.0)	0.0 (0.0–77.5)	<0.001	0.003	0.951^**^	0.0 (0.0–33.3)	58.3	78.6	91.7	96.4
Kebabs *“Ćevapi”*	84	188.0 (99.3–282.3)	172.0 (87.0–313.5)	0.0 (−16.0 to 16.0)	0.028	0.053	0.975^**^	0.0 (−9.1 to 11.1)	48.8	89.3	100.0	–
Walnut roll	76	150.0 (115.3–197.0)	170.0 (130.0–210.5)	6.7 (0.0–13.5)	<0.001	0.003	0.983^**^	4.7 (0.0–8.5)	78.9	94.7	100.0	–
Poppy seed roll	76	158.0 (115.7–200.3)	172.0 (130.0–214.5)	0.0 (0.0–14.2)	0.058	0.100	0.968^**^	0.0 (0.0–7.1)	78.9	92.1	100.0	–
Dumplings stuffed with plum	76	238.0 (120.0–494.3)	298.0 (142.0–547.5)	0.0 (0.0–22.0)	0.026	0.052	0.988^**^	0.0 (0.0–10.8)	57.9	94.7	98.7	100.0
Custard slice *“Kremšnita”*	84	162.7 (109.7–252.3)	174.5 (117.0–210.0)	0.0 (0.0–14.7)	0.026	0.052	0.934^**^	0.0 (0.0–11.8)	61.9	78.6	95.2	97.6
Pancakes	87	203.0 (125.0–396.0)	183.5 (125.0–357.5)	0.0 (−14.0 to 0.0)	0.999	0.999	0.991^**^	0.0 (−11.9 to 0.0)	62.1	87.4	96.6	100.0

Data are presented as median (interquartile range) or percentages. Difference (*g*) = estimate size − portion size; Relative difference (%) = ((estimated size − portion size)/portion size) × 100; FDR adjustment—Wilcoxon signed-rank test adjusted p values with the Benjamini-Hochberg false discovery rate procedure; “–” – all participants had within ± 50% differences or some of them had higher than ± 75% differences; ^**^p < 0.01.

Results from the Bland–Altman analysis (Supplementary Figures S1–S30) show that the acceptability of LOA width varied by dish type and portion size. Narrow LOA ranges, indicating higher precision, were observed for smaller portion sizes and dishes with countable food items. Wider LOA ranges were found for multicomponent dishes without clearly defined shapes and structure. Moreover, the analysis indicates that in 16 dishes, the dispersion of the difference between actual and estimated portion size was more pronounced when assessing larger portions, while in 6 dishes it was more pronounced when assessing medium portions. No recognizable pattern of distribution was observed in 8 dishes.

In 20 dishes, more than half of the portion size estimations were correct compared with the actual portion size (Table 3). A high proportion (>80%) of correct estimations was found for grilled fish with Swiss chard, kebabs *“Ćevapi,”* dumplings stuffed with plum, and pancakes. The proportion of distant estimations ranged from 0.0 to 31.0%. Dry spice cured sausage *“Kulen,”* cottage cheese with sour cream, brown roux soup *“Prežgana juha,”* roasted turkey with pasta *“Mlinci,”* roasted lamb with potatoes, and kebabs *“Ćevapi”* in flatbread *“Lepinja”* had more than 10% distant estimations, while octopus and potato cooked under the bell, spicy fish stew *“Fiš”* and Slavonian meat stew *“Čobanac”* had more than 20%. Quadratic weighted Cohen’s kappa analysis showed significant (*p* < 0.001) almost perfect agreement for 29 dishes, while Slavonian meat stew *“Čobanac”* demonstrated substantial agreement between assessments of actual and estimated portion sizes. Indeed, about 20–30% of participants found cottage cheese, dry spice-cured sausage *“Kulen,”* spicy fish stew *“Fiš,”* and brown roux soup *“Prežgana juha”* difficult to estimate, while for 11 dishes up to 10% of participants stated that it was very difficult to estimate their portion size (Figure 4).

**Table 3:** Distribution of reported portion size correct, adjacent or distant food picture when comparing it with serving size of dishes from food photography atlas of Croatian traditional foods.

Dishes	Observations	Percentage of participants			Cohen’s Kappa
	(*n*)	Correct	Adjacent (±1)	Distant (>±1)	*κ_w_*
Cheese “*Škripavac”*	76	40.8	55.3	3.9	0.931^**^
Dry-cured ham Prosciutto	76	56.6	34.2	9.2	0.936^**^
Dry spice cured sausage “*Kulen”*	76	40.8	46.1	13.2	0.904^**^
Bacon (diced)	76	64.5	31.6	3.9	0.954^**^
Bacon (sliced)	76	53.9	36.8	9.2	0.924^**^
Omelette with asparagus	84	41.7	51.2	7.1	0.926^**^
Baked cheese pastry *“Štrukli”*	87	51.7	39.1	9.2	0.939^**^
Cottage cheese with sour cream	76	46.1	38.2	15.8	0.906^**^
Phyllo pastry with cheese *“Burek”*	87	59.8	39.1	1.1	0.962^**^
Brown roux soup *“Prežgana juha”*	76	42.1	44.7	13.2	0.904^**^
Grilled fish with Swiss chard	84	86.9	10.7	2.4	0.982^**^
Fried small fish	84	41.7	48.8	9.5	0.925^**^
Risotto with squid	84	53.6	35.7	10.7	0.908^**^
Octopus and potato cooked under the bell	84	31.0	41.7	27.4	0.816^**^
Spicy fish stew *“Fiš”*	84	34.5	39.3	26.2	0.810^**^
Bean stew with smoked meat	87	55.2	41.4	3.4	0.933^**^
Roasted turkey with pasta *“Mlinci”*	87	51.7	35.6	12.6	0.919^**^
Dalmatian beef stew *“Pašticada”* with gnocchi	87	35.6	39.1	25.3	0.811^**^
Slavonian meat stew *“Čobanac”*	87	39.1	29.9	31.0	0.722^**^
Cooked vegetables *“Sataraš”* with roasted meat	84	61.9	34.5	3.6	0.940^**^
Roasted lamb with potatoes	87	51.7	32.3	16.1	0.919^**^
Stuffed pepper with mashed potatoes	87	58.6	39.1	2.3	0.946^**^
Stuffed cabbage rolls *“Sarma”* with mashed potatoes	87	52.9	37.8	9.2	0.920^**^
Kebabs *“Ćevapi”* in flatbread *“Lepinja”*	84	65.5	19.0	15.5	0.935^**^
Kebabs *“Ćevapi”*	84	81.0	14.3	4.8	0.967^**^
Walnut roll	76	65.8	34.2	0.0	0.969^**^
Poppy seed roll	76	67.1	30.3	2.6	0.963^**^
Dumplings stuffed with plum	76	89.5	9.2	1.3	0.988^**^
Custard slice *“Kremšnita”*	84	61.9	33.3	4.8	0.918^**^
Pancakes	87	86.2	13.8	0.0	0.988^**^

Data are presented as percentages; Quadratic weighted Cohen’s kappa (^**^p < 0.05).

**Figure 4:**
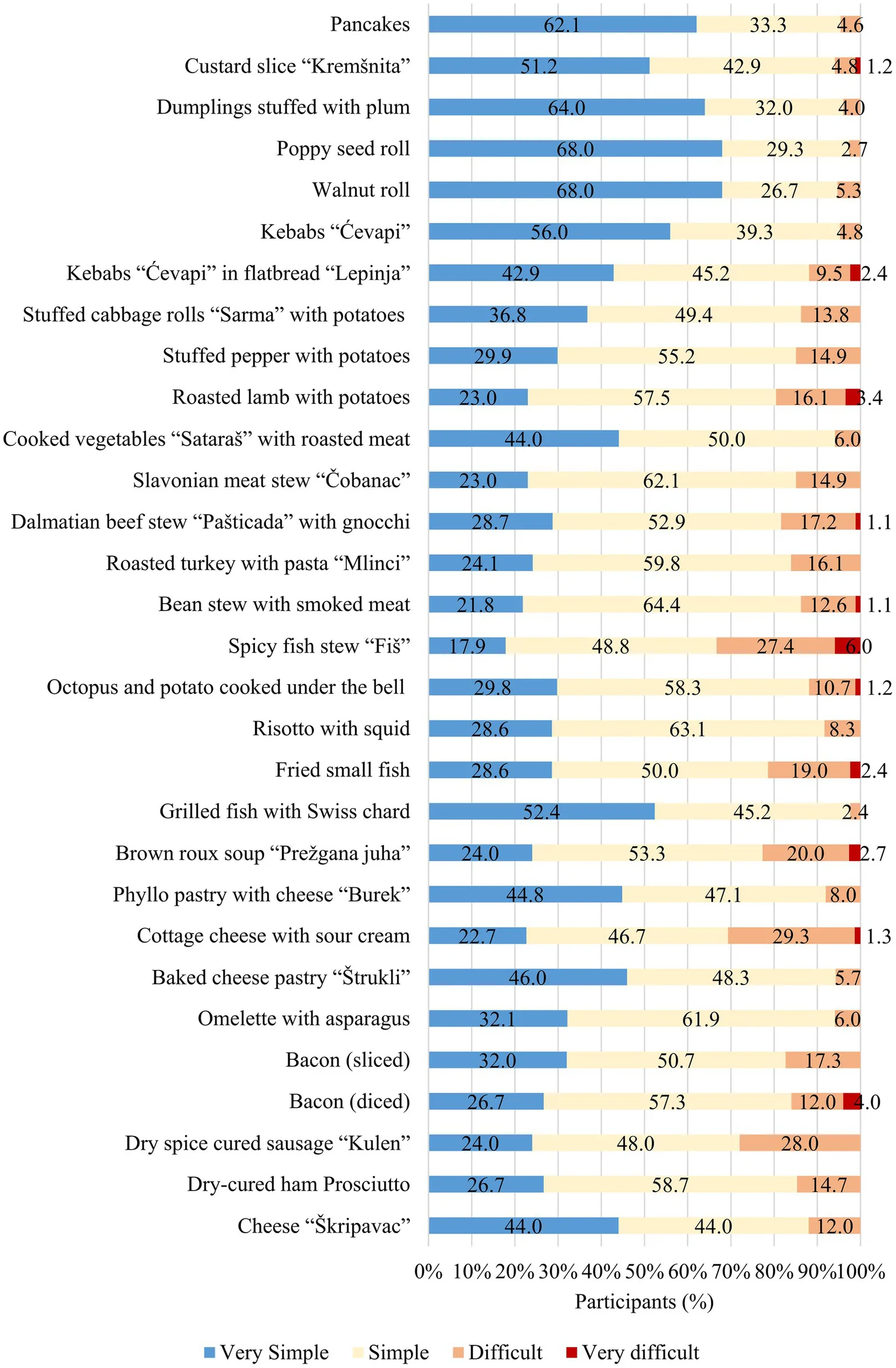
Distribution of participants according to perceived difficulty of the portion size estimation of dishes from food photography atlas of Croatian traditional foods.

## 4 Discussion

This is the first study to develop and validate a food photography atlas for portion size estimation of traditional Croatian dishes in an adult population. Previous regional food photographs have been developed to estimate portion sizes only for children (20) or have been designed for adults with three dish sizes (small, medium, large) that have not undergone formal statistical validation (27). There is also a validated food atlas for the Balkan region for adults, however, it includes only individual dishes and does not include Croatian national dishes (28). The results of this study, including the low relative difference and substantial to almost perfect agreement between actual and estimated serving sizes, support the validity of the atlas as a portion-size estimation aid.

In the present study, relative differences ranged from −23.2 to 13.9%, which is comparable to those reported in previous validation studies (8, 12, 29). However, in the previous literature, smaller ranges of relative differences have been reported when validating food photographs by Turconi et al. (−1.3 to 23.2%), Bouchoucha et al. (−4.5 to 18.6%), Huybregts et al. (−6.4 to 6.3%), Ding et al. (−14.71 to 5.56%), and Flax et al. (−14 to 10%) (7, 9, 30–32), while three studies reported larger ranges, which were particularly notable for overestimating portion sizes (11, 14, 33). These differences may be due to several methodological factors, such as the characteristics of the study population—for example, including only women or using a wide age range from children to adults (5, 15). Furthermore, there are variations in the number of photographs within a set for a particular dish. Specifically, using an odd number of photographs is considered to increase error in selecting the mean value, while too many images can lead to difficulties in decision-making (5). Additionally, during the validation phases of the studies, various methods were used to determine actual portion sizes, such as comparison with previously weighed food, food eaten the previous day or at the same day before validation, and dietary records. The selection of the instrument for validation depends on whether which type of food photographs were evaluated, e.g., linked to 24-h recall, digitized food photographs, or a printed food photography atlas (6, 10, 33, 34).

In this study significant underestimations were observed in octopus and potato cooked under the bell, omelette with asparagus, risotto with squid, spicy fish stew *“Fiš,”* Slavonian meat stew *“Čobanac”* and brown roux soup *“Prežgana”* in contrast overestimations were found in cottage cheese with sour cream and bean stew with smoked meat. The defining characteristic of all these dishes is that they are amorphous foods without clearly defined shapes. Indeed, it has already been shown that amorphous dishes make it more difficult to estimate portion sizes (7, 9, 14, 35). However, for stuffed cabbage rolls *“Sarma”* with mashed potatoes the difference between actual and estimated size was significant but with low median difference. This may be due the fact that stuffed cabbage rolls *“Sarma”* can be counted, which makes it easier to assess portion size (12). Moreover, other amorphous dishes that contained some countable food items (e.g., grilled fish with Swiss chard, roasted turkey with pasta *“Mlinci,”* Dalmatian beef stew *“Pašticada”* with gnocchi, cooked vegetables *“Sataraš”* with roasted meat, roasted lamb with potatoes and stuffed pepper with mashed potatoes) did not show a significant difference between actual and estimated portion sizes. The strong Spearman correlations indicated that participants generally preserved the ranking of portion sizes, with larger actual portions being estimated as larger which is in line with existing literature (10, 34). However, correlation does not indicate how closely the estimated values agreed with the actual portion sizes; therefore, agreement was evaluated separately using Bland–Altman analysis and weighted Cohen’s kappa.

Based on visual assessment using Bland–Altman plots, half of the dishes had a median difference of 0.0 with narrow and acceptable LOA relative to their typical portion size range, indicating that respondents overestimated and underestimated portion sizes to a similar extent, resulting in low overall systematic bias. Particularly narrow LOAs were observed in dishes that contained single-unit food items or other countable food items. However, Bland–Altman plots also revealed inconsistencies, including both overestimation and underestimation in medium and large portions. In dishes such as cottage cheese with sour cream, bean stew with smoked meat and kebabs *“Ćevapi”* in flatbread *“Lepinja”* overestimation was observed in larger portions, while in dishes such as risotto with squid, octopus and potato cooked under the bell, spicy fish stew “*Fiš,”* omelette with asparagus and kebabs *“Ćevapi”* underestimation was observed in larger portions. These results do not support previous studies that have identified the flat-slope phenomenon, in which larger portions are systematically underestimated and smaller portions are overestimated (7, 12, 13, 29, 33). Moreover, greater dispersion was observed in six dishes with medium portion sizes, while there was no clear evidence of greater dispersion in estimation errors for smaller portion sizes. This suggests that larger amounts of food and more visual clutter on the plate can influence portion size estimates. Moreover, larger portions in previous studies were associated with less agreement and greater discrepancies (6, 9, 12, 36).

The proportion of correctly identified portion sizes varied across dishes, ranging from 31.0% for octopus and potatoes cooked under the bell to 89.5% for dumplings stuffed with plum. However, when adjacent estimations were also included, the proportion of acceptable estimations exceeded 70% for 11 dishes and 90% for 10 dishes. Overall, the proportion of distant estimations ranged from 0.0 to 31.0%. The dishes with the highest proportions of distant estimations were Slavonian meat stew *“Čobanac”* (31.0%), octopus and potatoes cooked under the bell (27.4%), spicy fish stew *“Fiš”* (26.2%), and Dalmatian beef stew *“Pašticada”* with gnocchi (25.3%). Accordingly, lower but still substantial agreement, assessed with quadratic Cohen’s kappa, was observed for these dishes. Moreover, the participant stated that these dishes were difficult to assess. All four dishes had irregular or liquid shapes, which may make estimation more difficult. In contrast, foods with a clearly defined shapes and countable food items showed better level of agreement. Similar patterns have been reported in previous studies, but with lower values of *κ_w_* (6, 8). Higher *κ_w_* values indicate better agreement in portion size estimation, reducing errors in estimating energy and nutrient intakes (7, 10, 31).

Overall, based on the primary and secondary criteria for acceptable agreement, the food photography atlas for portion size estimation of traditional Croatian dishes in adults demonstrates acceptable validity and utility for dietary assessment. These criteria were established in line with previous literature (8, 11, 28, 31, 34). The primary criteria were met in all 30 dishes, confirming that the food photography atlas accurately ranks and categorizes portion sizes of traditional dishes into the correct intake levels. Furthermore, 29 of 30 dishes met the secondary criteria for the precision threshold, with more than 50% of estimates falling within ±25% of the actual portion size. Relative difference was higher than 20% in only one dish: octopus and potato cooked under the bell. Wider Bland–Altman LOA and patterns of overestimation and underestimation in medium and large portions were mainly observed in amorphous, multicomponent dishes without clearly defined shapes.

This food photography atlas for portion size estimation of traditional Croatian dishes was developed for an adult population. Accordingly, a sample of 110 adults participated in the validation phase of the study. It should be emphasized that the sample included more women and a highly educated population, which could have contributed to better portion size estimates (15, 36). In addition, three-quarters of participants had professions unrelated to food, so we ensured that the general population could recognize portion sizes and use the food photography atlas (15). Although the sample was not consistent across all 3 days of the study, each dish was validated with an adequate sample size. A standardized protocol was used to define portion sizes and prepare the photographs (5). Moreover, the portion sizes were estimated from the Croatian national study of adults’ eating habits (21). Validation was conducted in laboratory experiments, where the food was served on the same plates, which may have contributed to more accurate assessments. Some of the dishes used for photography and validation were not ordered from the same restaurants, which could lead to greater errors during validation (33).

In conclusion, the food photography atlas showed generally acceptable agreement for estimating portions of traditional Croatian dishes under controlled laboratory conditions. However, for several dishes, greater error was observed in the assessment of larger portions, which should be taken into account when using the food photography atlas. This suggests that the food photography atlas of traditional Croatian dishes could be an accurate tool for estimating portion sizes in the adult population of Croatia.

## Data Availability

The datasets presented in this article are not readily available because they belong to the Croatian Agency for Agriculture and Food. Requests to access the datasets should be directed to both the Croatian Agency for Agriculture and Food and the corresponding author.

## Author’s note

Tea Knezović and Ana Jurčić are students of graduated nutrition study.
