# Supplementary material for: Development and validation of a food photography atlas for portion size estimation of traditional Croatian dishes in adults
Source: Front Nutr. 2026 Oct 7;13:1956906. doi: 10.3389/fnut.2026.1956906 (PMC13683142; doi:10.3389/fnut.2026.1956906)
Supplement: Supplementary file 1 [file Data_Sheet_1.PDF]

Supplementary Material

Supplementary Table 1. Portion sizes of the dishes from the food photography atlas of traditional Croatian dishes for adults

Dishes	Percentiles (g)			
	5th	35th	65th	95th
Cheese “*Škripavac*”	26.0	46.0	129.0	230.0
Dry-cured ham Prosciutto	22.5	52.5	75.0	172.5
Dry spice cured sausage “*Kulen*”	27.0	54.0	126.0	191.0
Bacon (diced)	14.0	64.0	116.0	183.0
Bacon (sliced)	18.0	62.0	116.0	185.0
Omelette with asparagus	58.0	173.0	247.0	444.0
Baked cheese pastry *“Štrukli”*	96.0	184.0	270.0	385.0
Cottage cheese with sour cream	34.0	107.0	177.0	355.0
Phyllo pastry with cheese *“Burek”*	75.0	138.0	335.0	631.0
Brown roux soup *“Prežgana juha”*	157.5	195.0	262.5	337.5
Grilled fish with Swiss chard	217.0	446.5	803.0	1118.0
Fried small fish	79.0	236.0	355.0	537.0
Risotto with squid	140.0	290.0	406.0	480.0
Octopus and potato cooked under the bell	115.0	237.0	415.0	570.0
Spicy fish stew *“Fiš”*	210.0	270.0	360.0	480.0
Bean stew with smoked meat	160.0	330.0	461.5	745.0
Roasted turkey with pasta *“Mlinci”*	147.0	332.0	438.0	626.0
Dalmatian beef stew *“Pašticada”* with gnocchi	277.0	420.0	512.0	829.0
Slavonian meat stew *“Čobanac”*	230.0	323.0	395.0	485.0
Cooked vegetables *“Sataraš”* with roasted meat	147.0	313.0	487.0	751.0
Roasted lamb with potatoes	192.0	342.0	515.0	674.0
Stuffed pepper with mashed potatoes	240.0	389.0	598.0	965.0
Stuffed cabbage rolls *“Sarma”* with mashed potatoes	227.0	315.0	510.0	793.0
Kebabs *“Ćevapi”* in flatbread *“Lepinja”*	108.0	208.0	405.0	560.0
Kebabs *“Ćevapi”*	50.0	124.0	220.0	407.0
Walnut roll	86.0	130.0	170.0	251.0
Poppy seed roll	87.0	130.0	172.0	257.0
Dumplings stuffed with plum	76.0	208.0	388.0	707.0
Custard slice *“Kremšnita”*	95.0	139.0	210.0	337.0
Pancakes	41.0	125.0	242.0	473.0

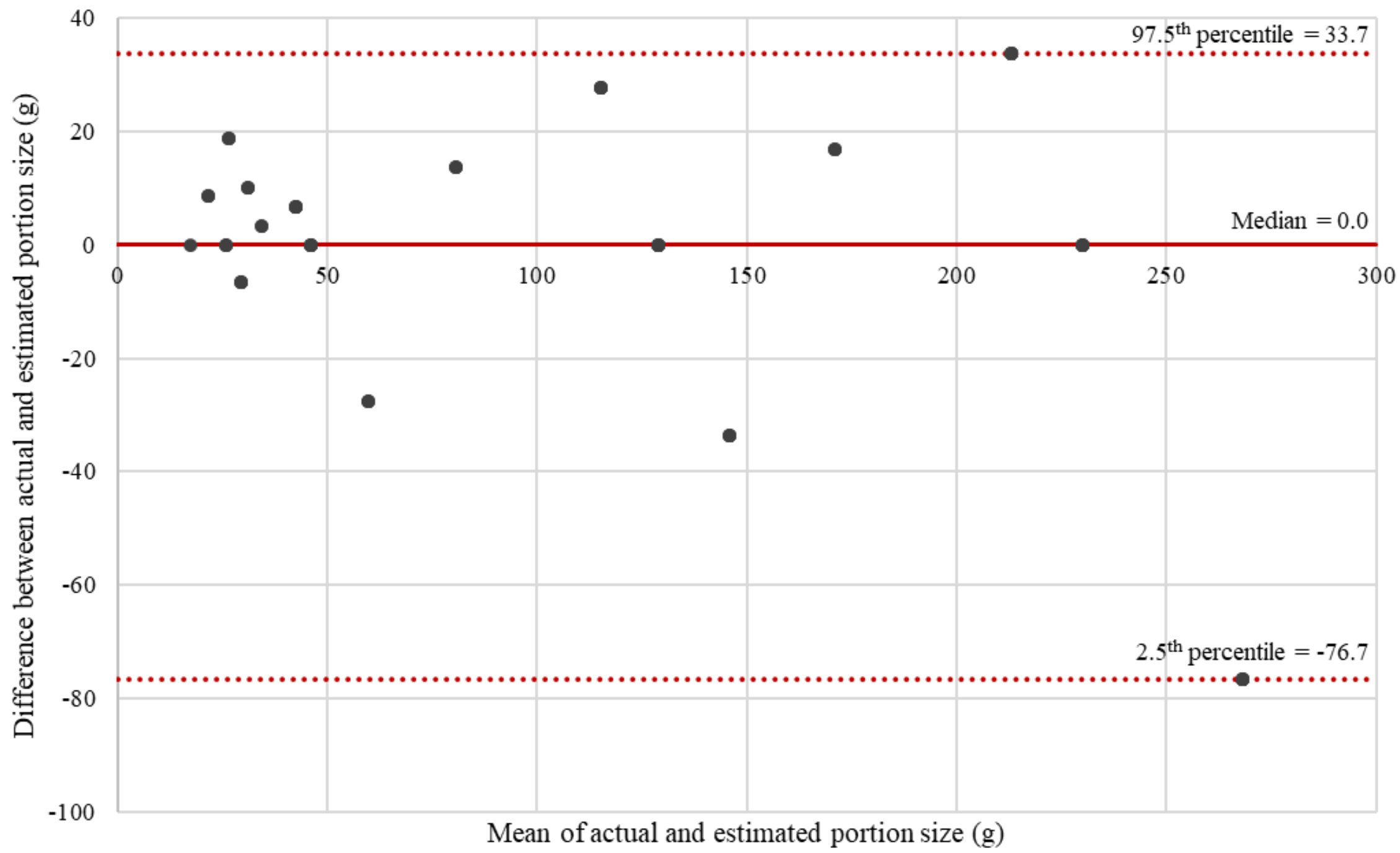

Supplementary Figure 1. Bland–Altman plots showing agreement between the actual and estimated portion size for Cheese *"Škripavac"*

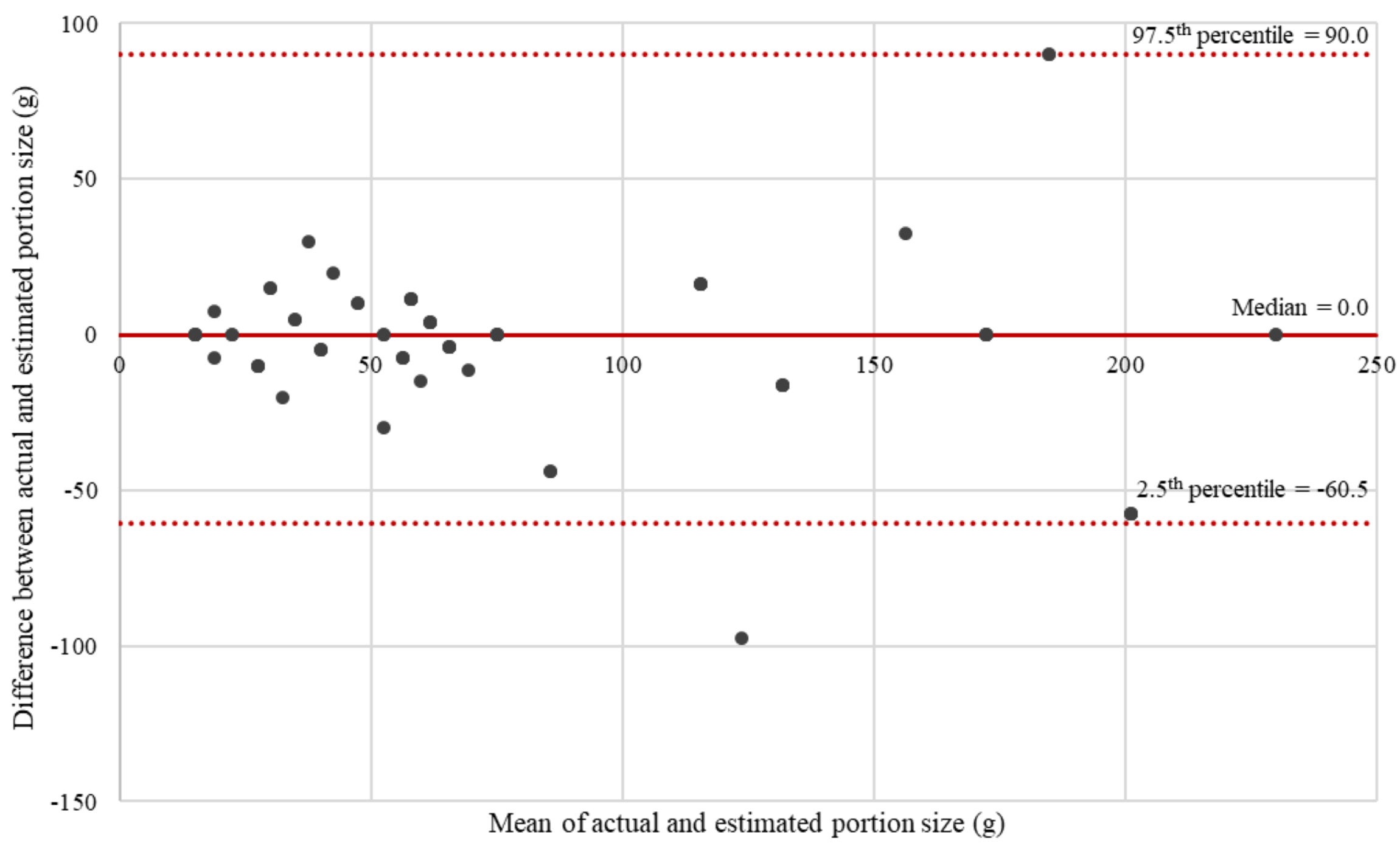

Supplementary Figure 2. Bland–Altman plots showing agreement between the actual and estimated portion size for dry-cured ham Prosciutto

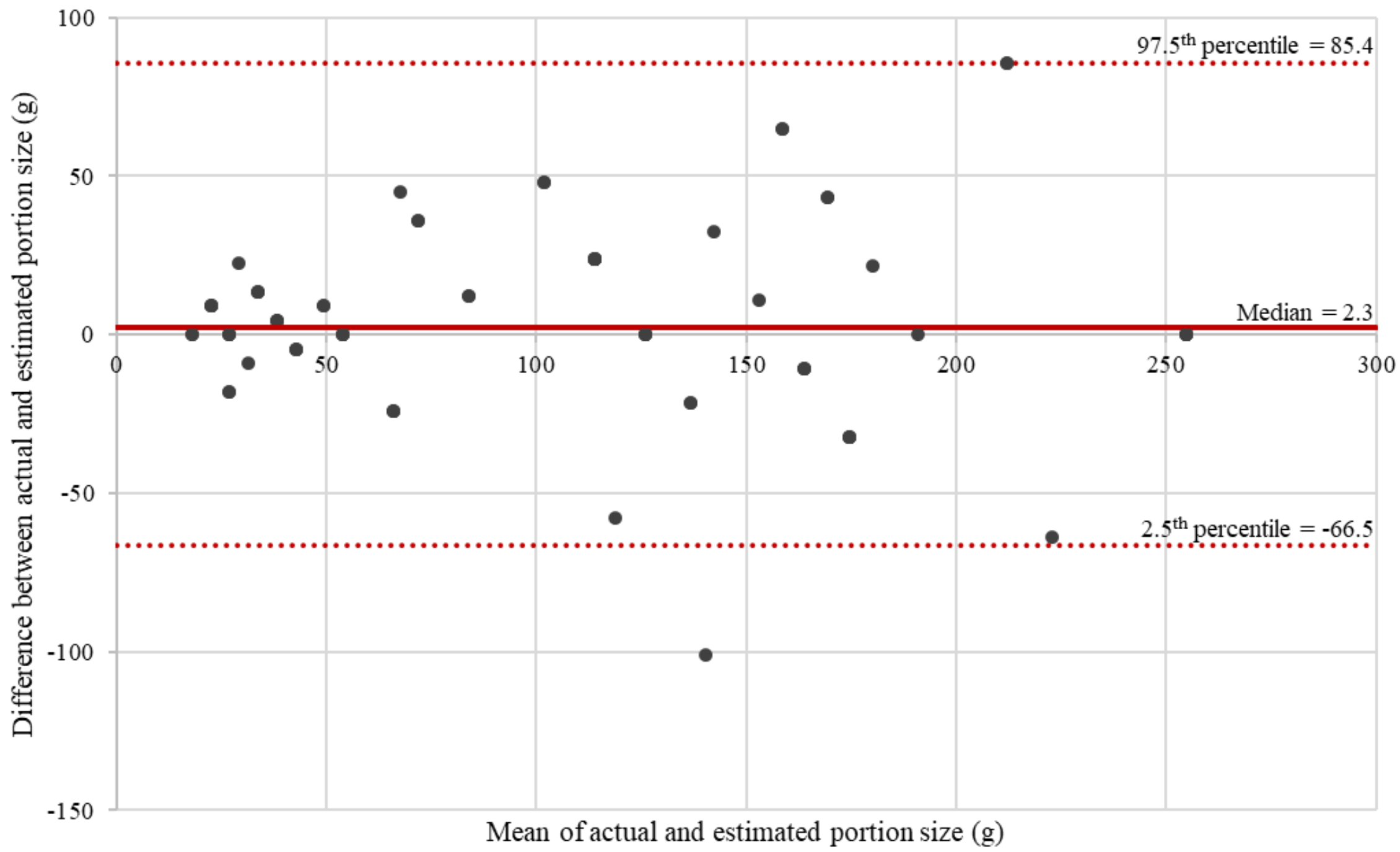

Supplementary Figure 3. Bland–Altman plots showing agreement between the actual and estimated portion size for dry spice cured sausage *"Kulen"*

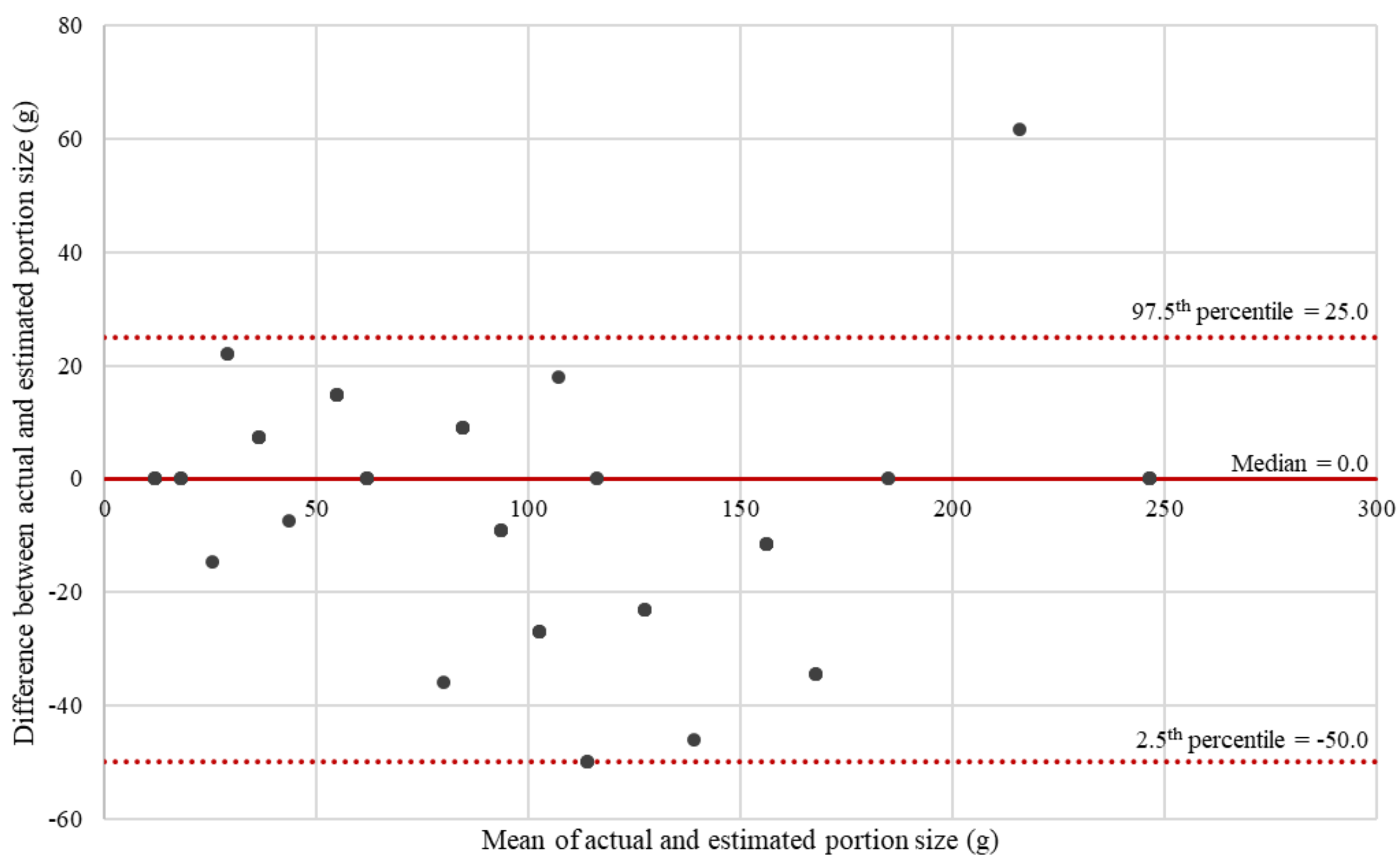

Supplementary Figure 4. Bland–Altman plots showing agreement between the actual and estimated portion size for diced bacon

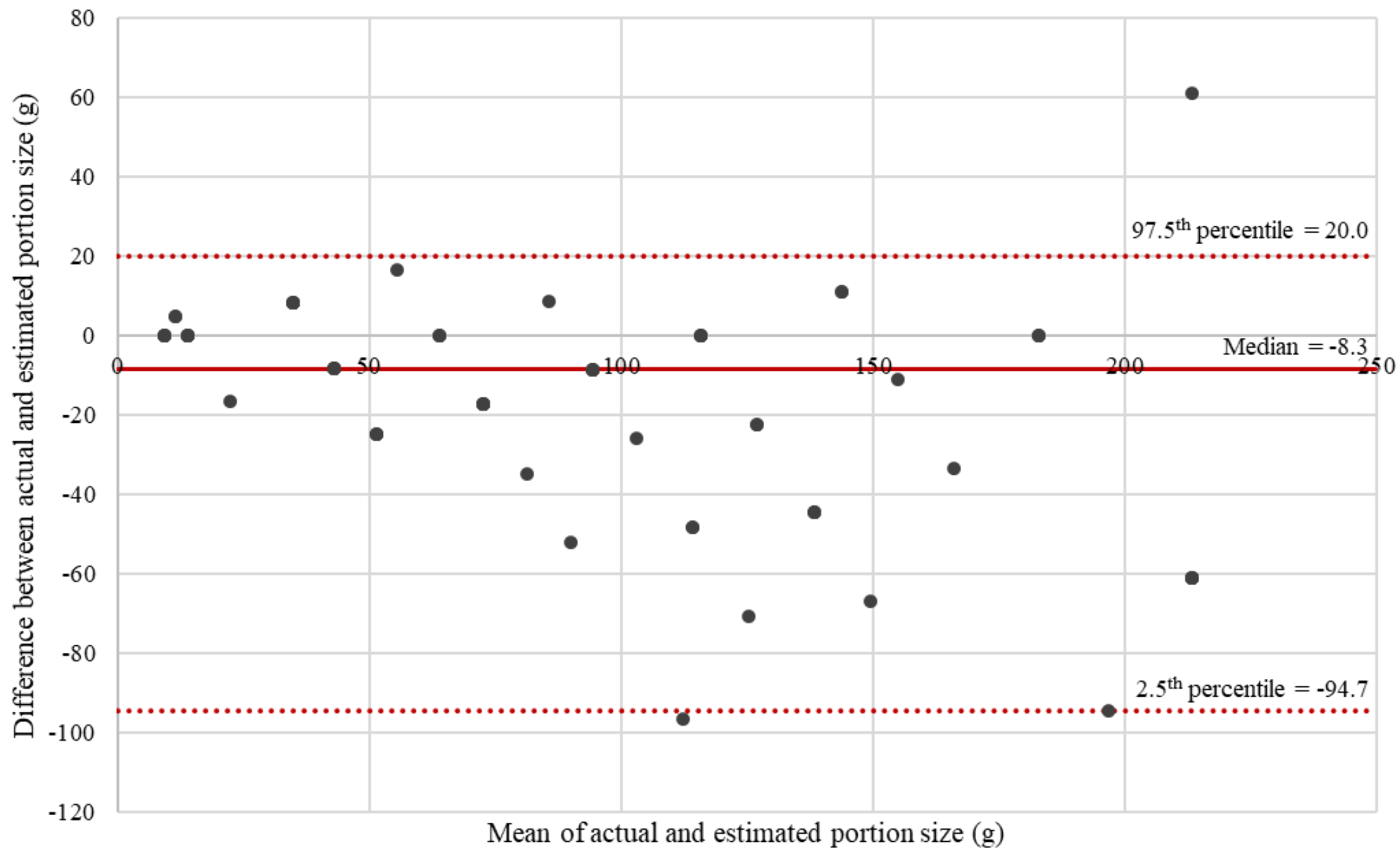

Supplementary Figure 5. Bland–Altman plots showing agreement between the actual and estimated portion size for sliced bacon

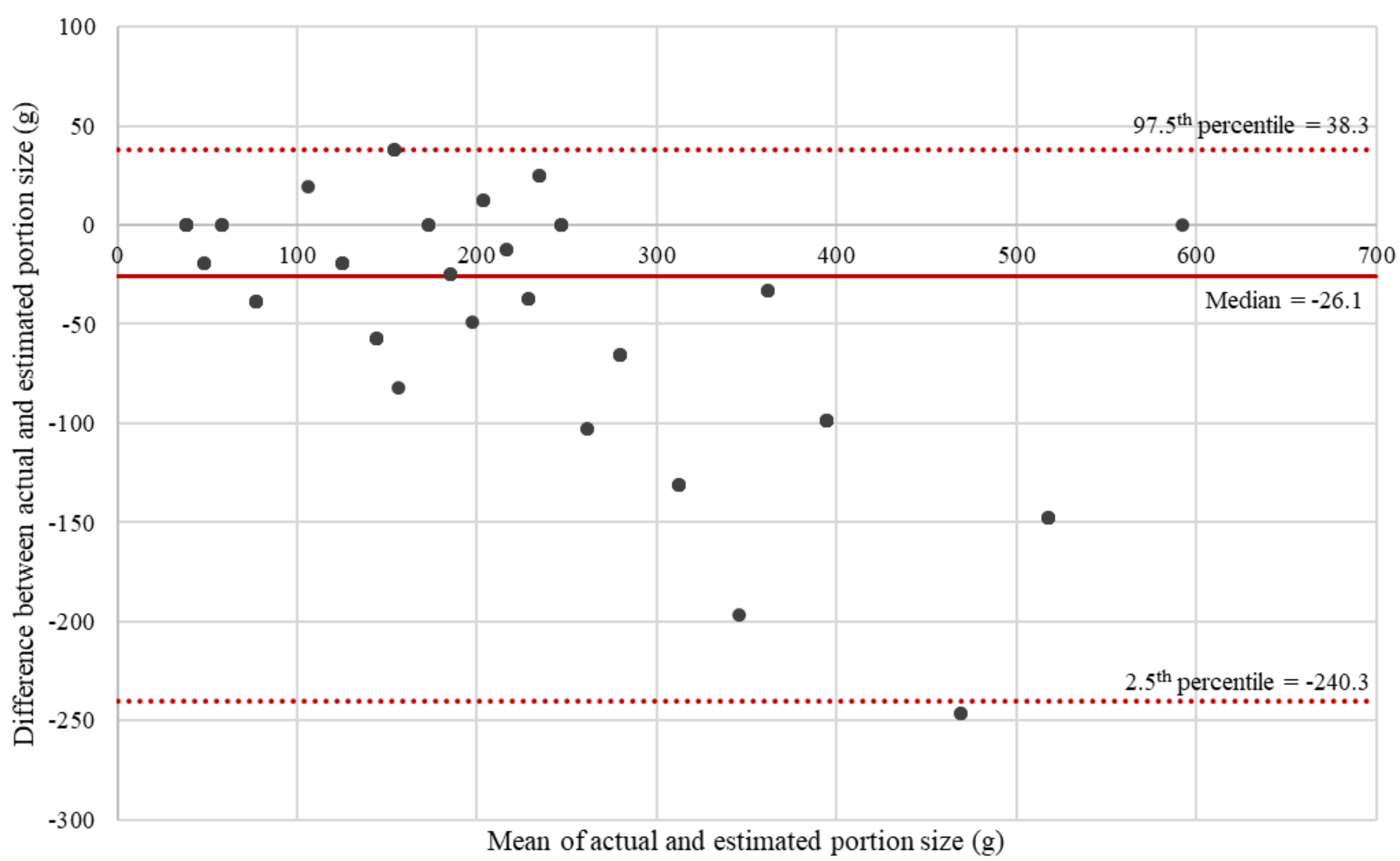

Supplementary Figure 6. Bland–Altman plots showing agreement between the actual and estimated portion size for omelette with asparagus

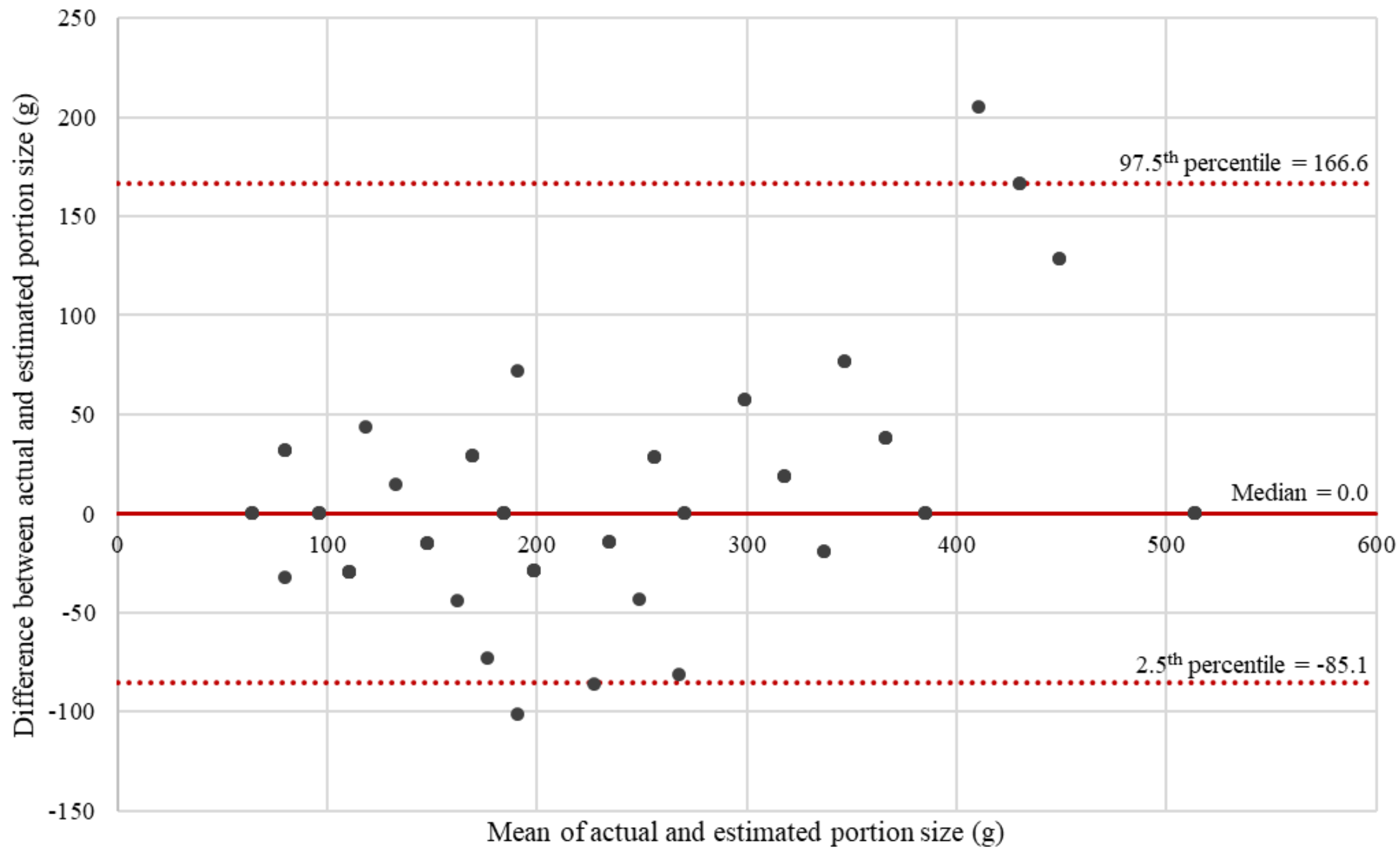

Supplementary Figure 7. Bland–Altman plots showing agreement between the actual and estimated portion size for baked cheese pastry *"Štrukli"*

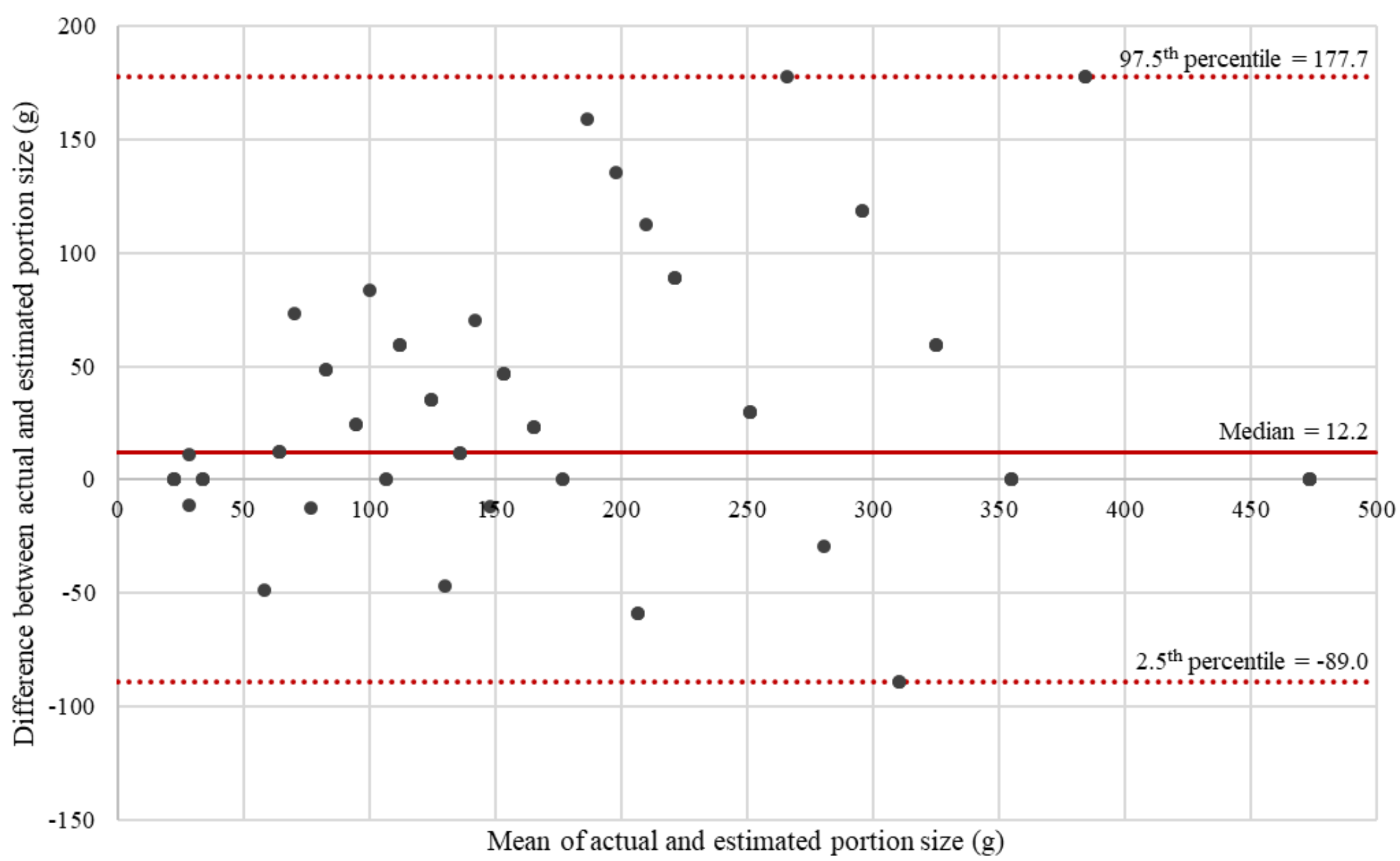

Supplementary Figure 8. Bland–Altman plots showing agreement between the actual and estimated portion size for cottage cheese with sour cream

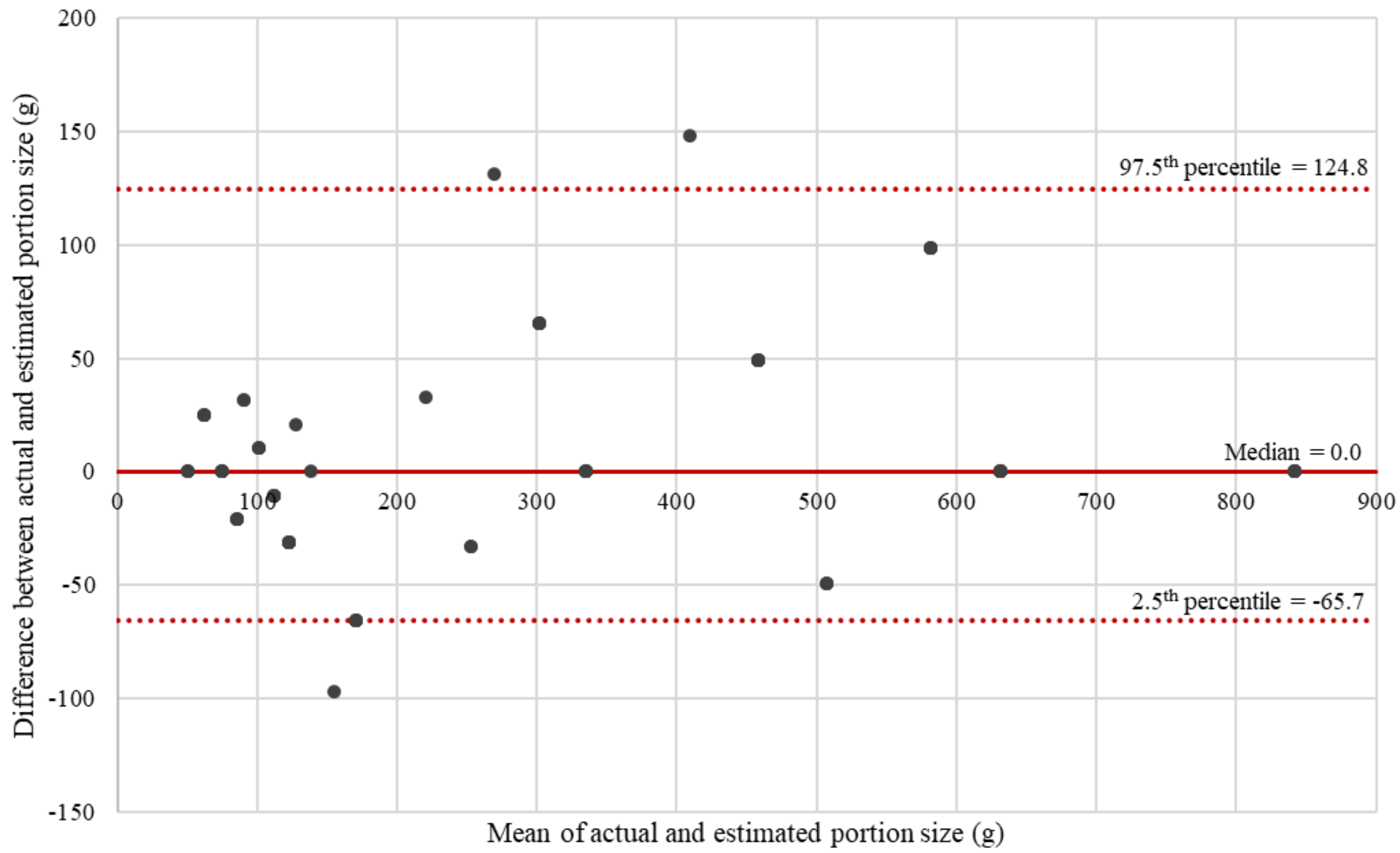

Supplementary Figure 9. Bland–Altman plots showing agreement between the actual and estimated portion size for phyllo pastry with cheese *"Burek"*

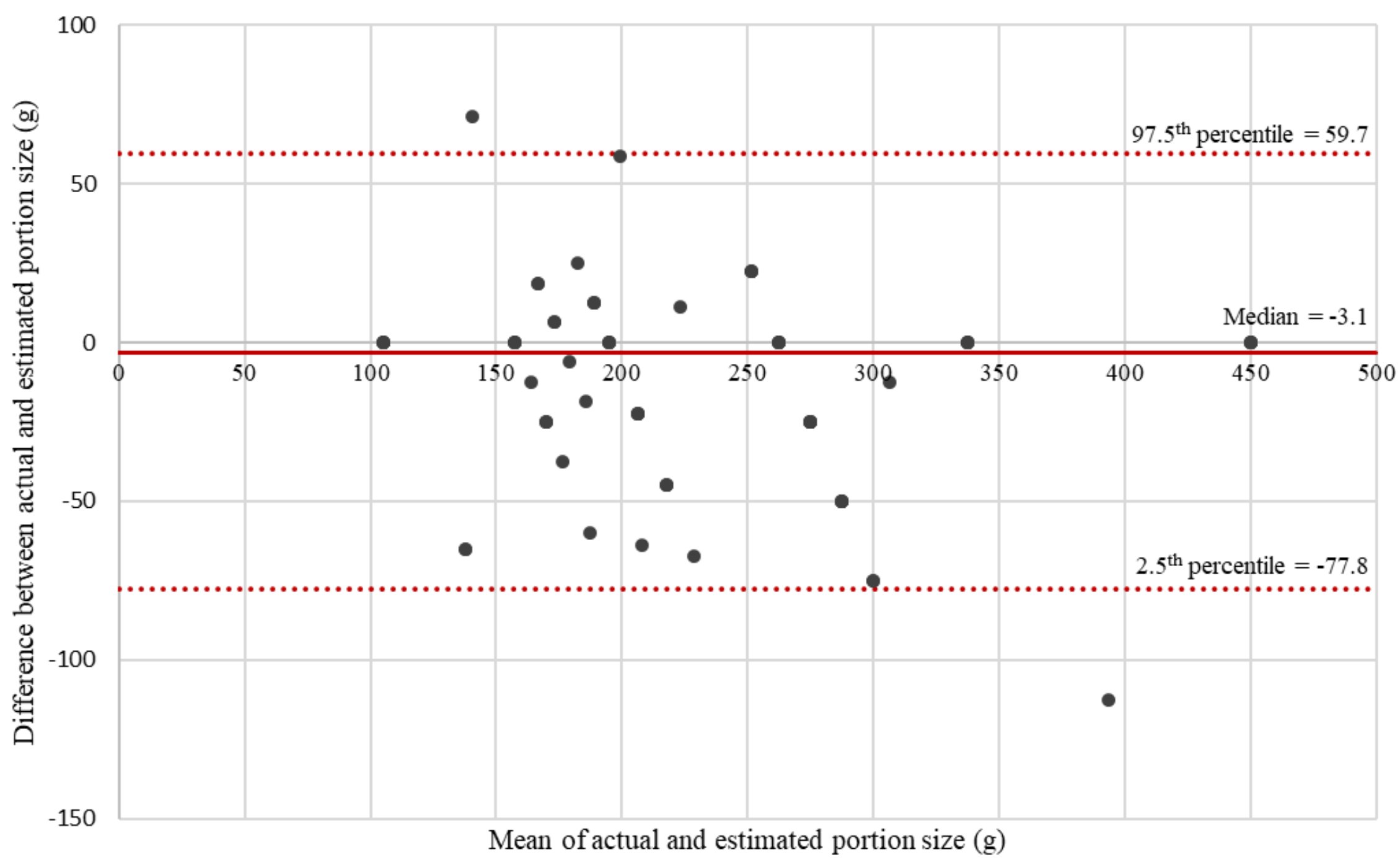

Supplementary Figure 10. Bland–Altman plots showing agreement between the actual and estimated portion size for brown roux soup *"Prežgana juha"*

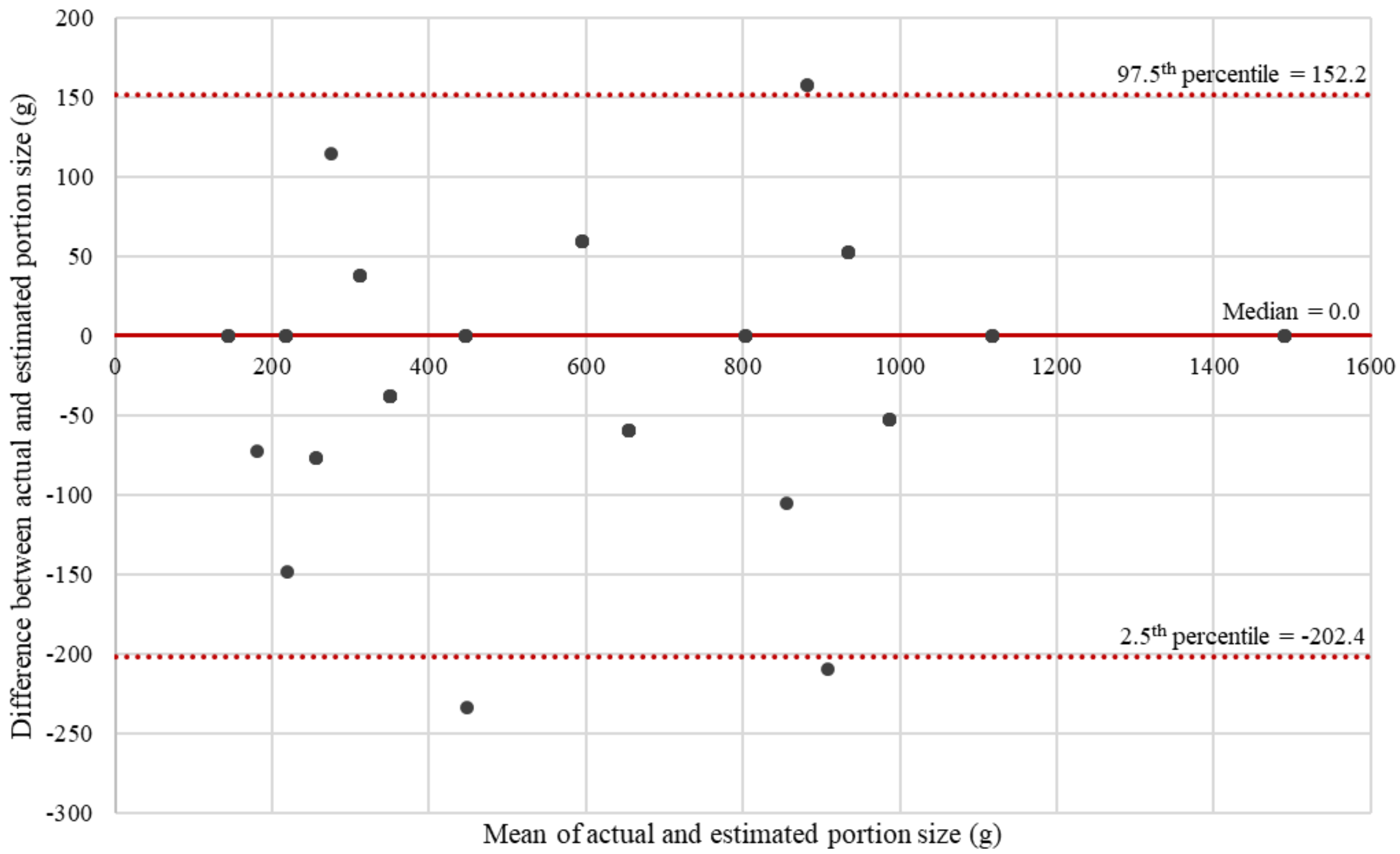

Supplementary Figure 11. Bland–Altman plots showing agreement between the actual and estimated portion size for grilled fish with Swiss chard

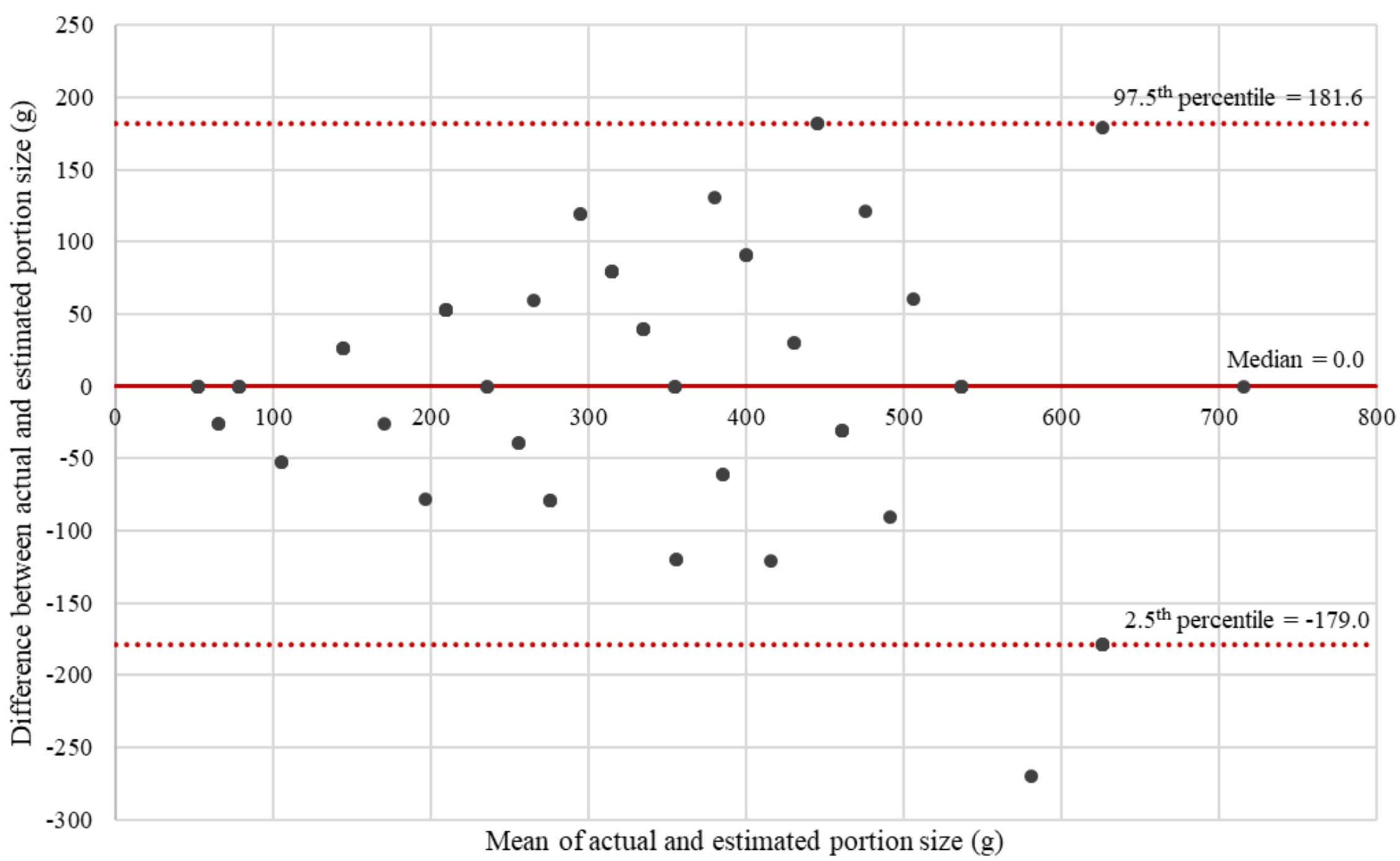

Supplementary Figure 12. Bland–Altman plots showing agreement between the actual and estimated portion size for fried small fish

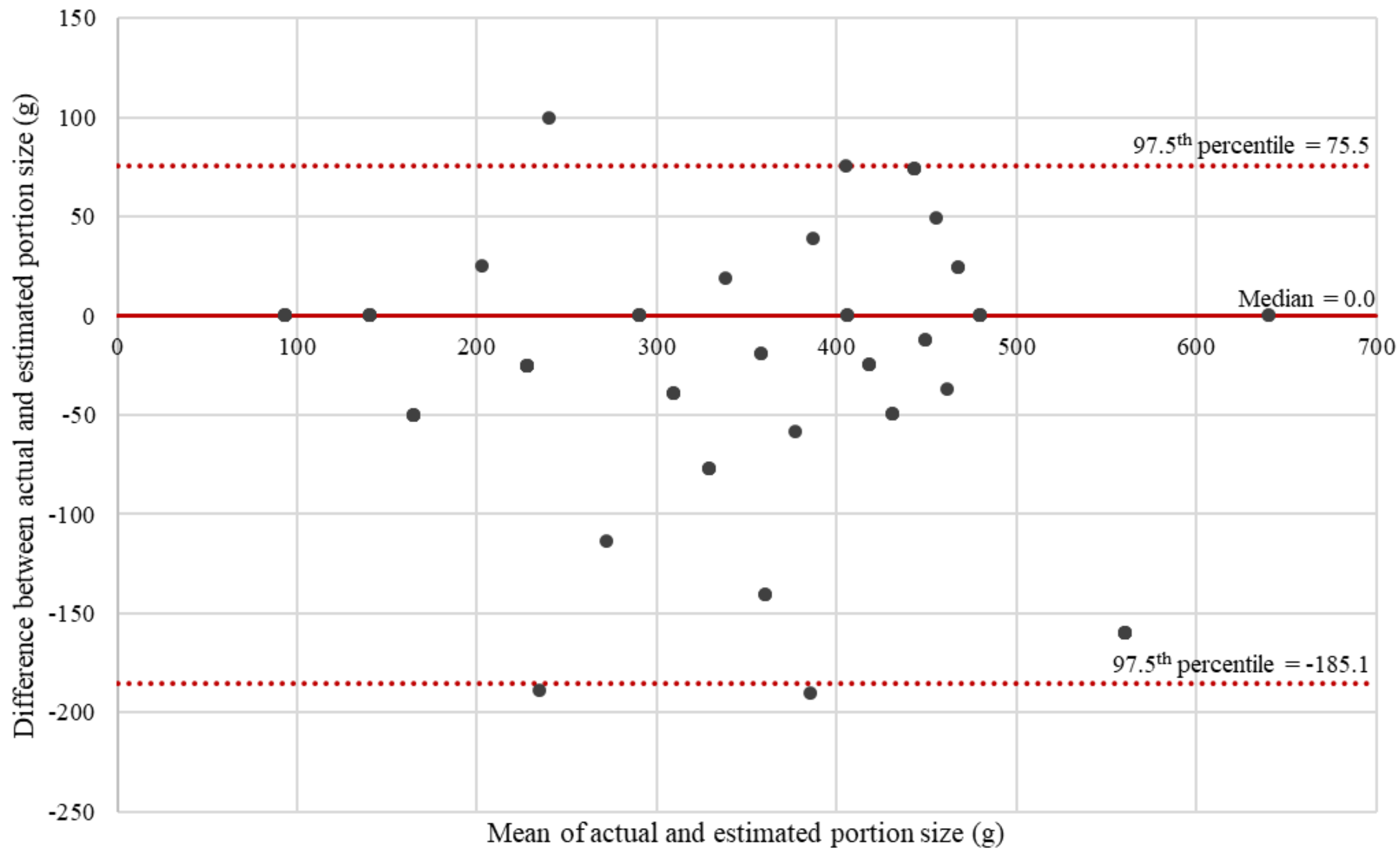

Supplementary Figure 13. Bland–Altman plots showing agreement between the actual and estimated portion size for risotto with squid

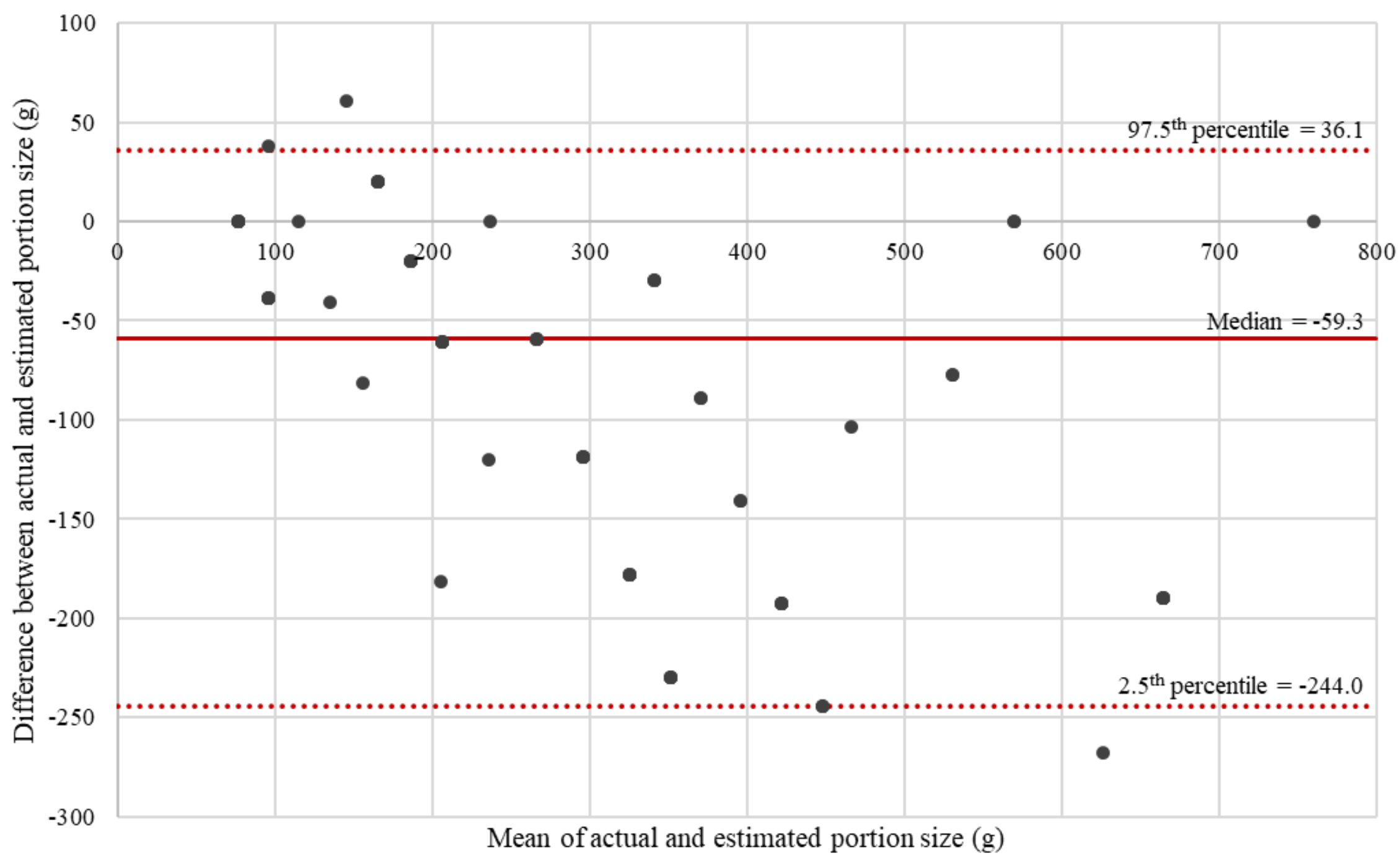

Supplementary Figure 14. Bland–Altman plots showing agreement between the actual and estimated portion size for octopus and potato cooked under the bell

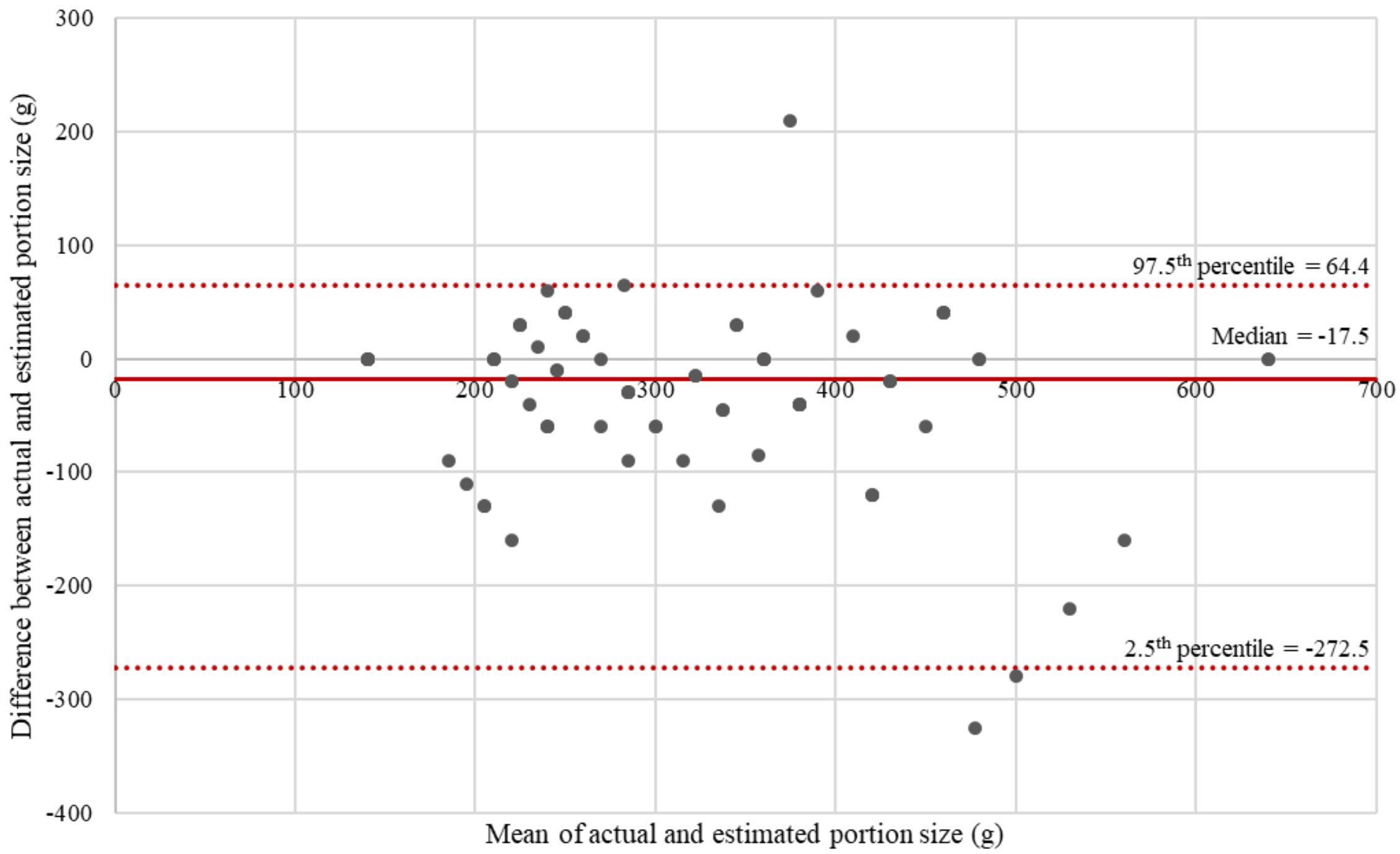

Supplementary Figure 15. Bland–Altman plots showing agreement between the actual and estimated portion size for spicy fish stew *"Fiš"*

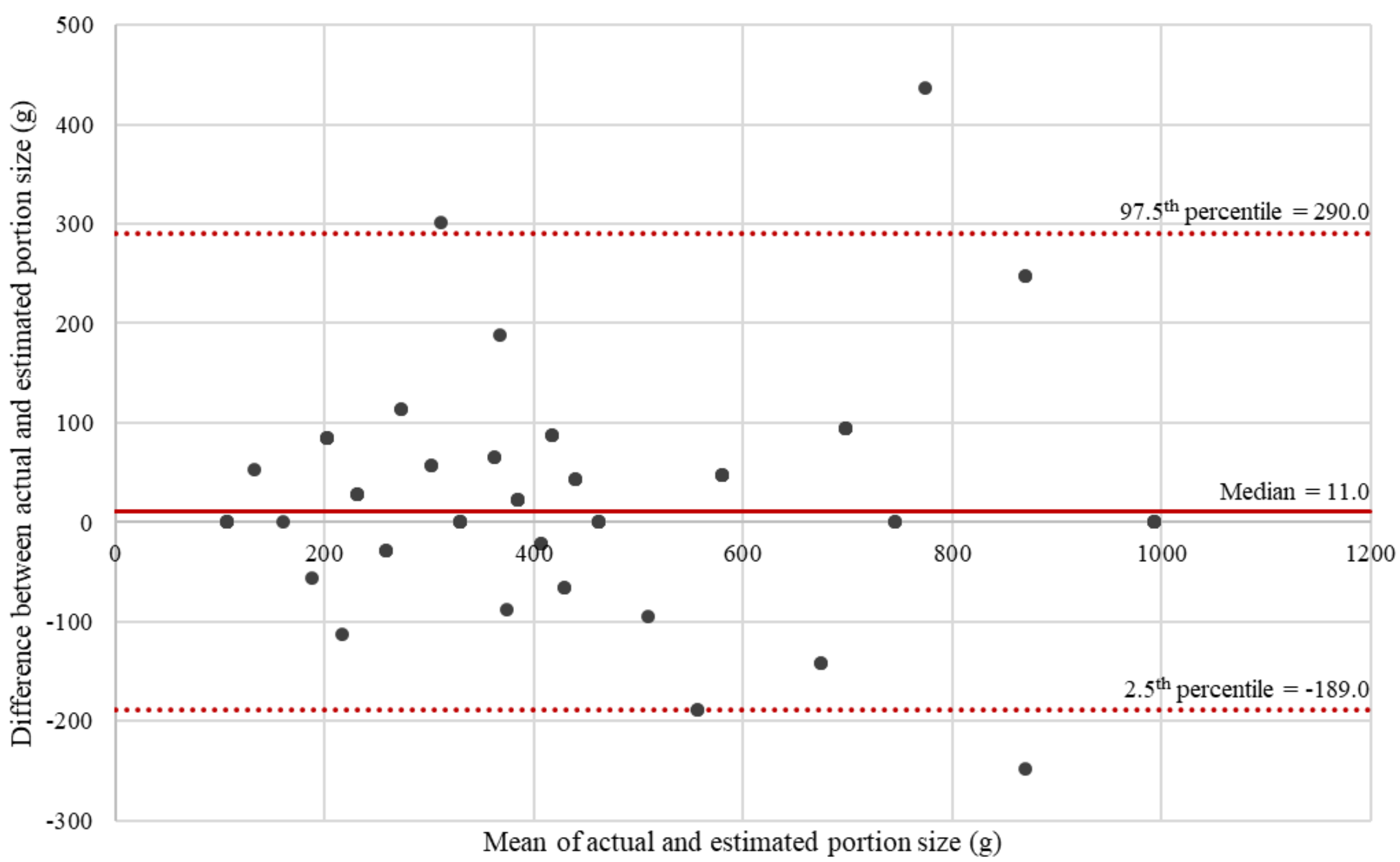

Supplementary Figure 16. Bland–Altman plots showing agreement between the actual and estimated portion size for bean stew with smoked meat

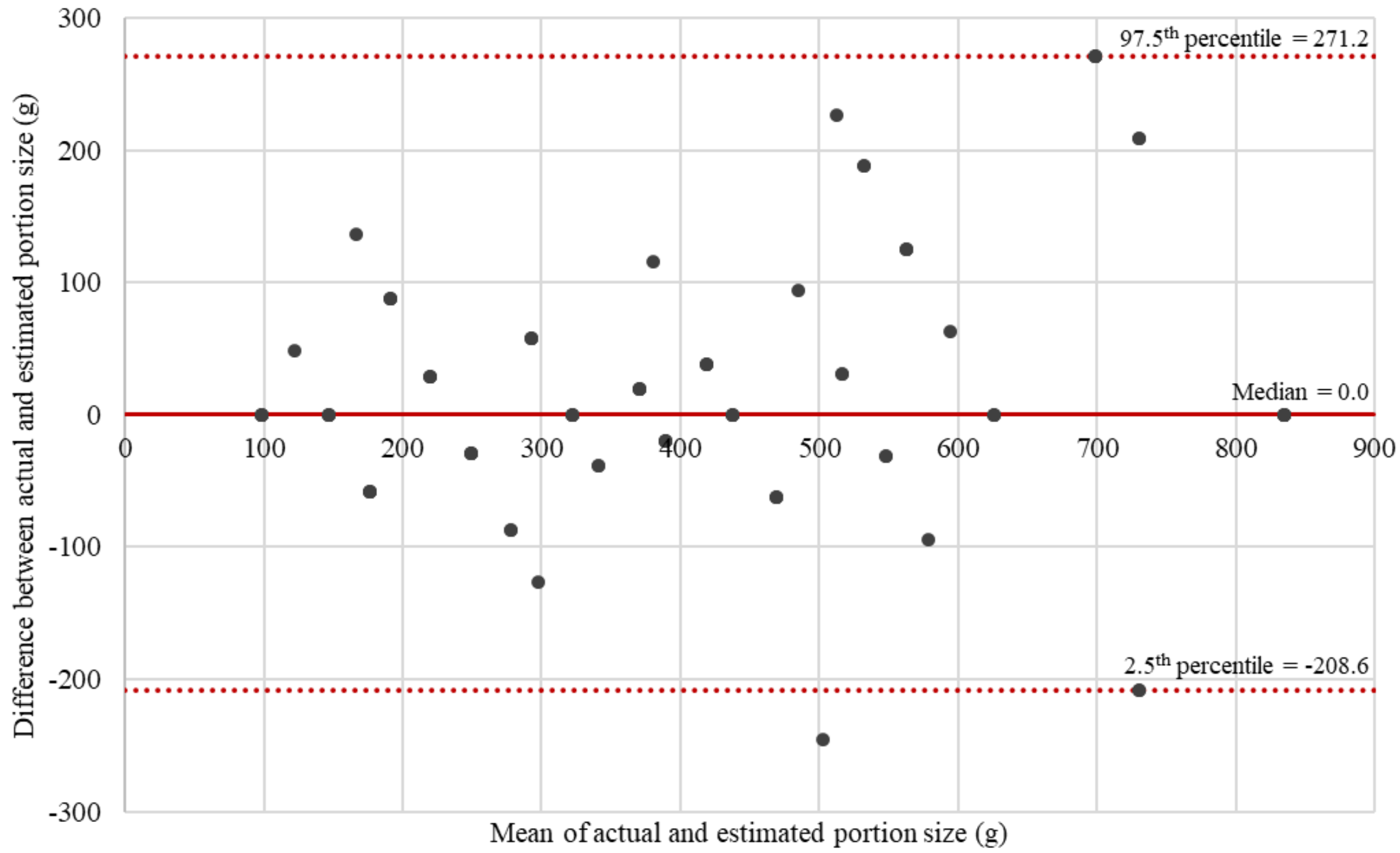

Supplementary Figure 17. Bland–Altman plots showing agreement between the actual and estimated portion size for roasted turkey with pasta *"Mlinci"*

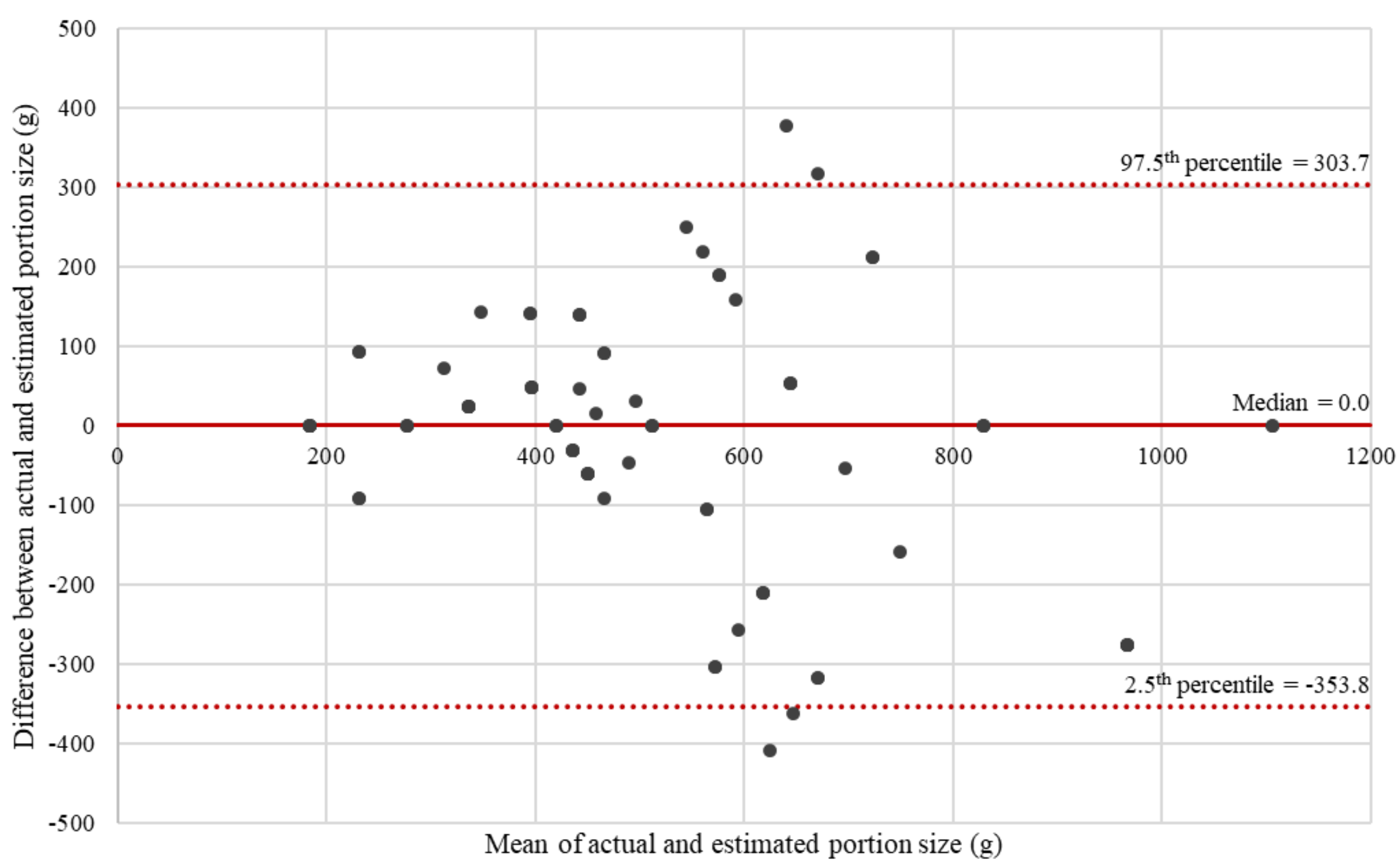

Supplementary Figure 18. Bland–Altman plots showing agreement between the actual and estimated portion size for Dalmatian beef stew *"Pašticada"* with gnocchi

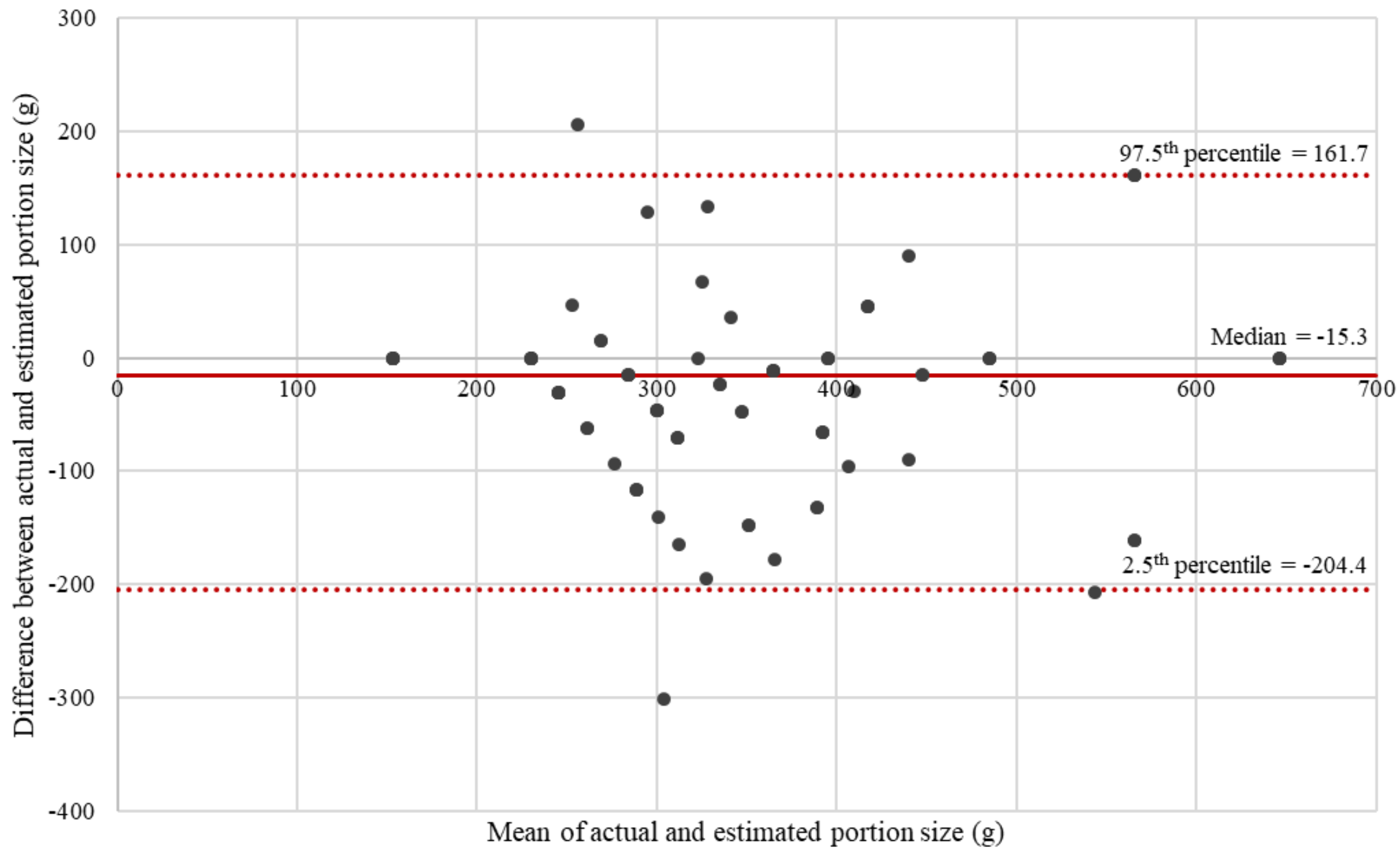

Supplementary Figure 19. Bland–Altman plots showing agreement between the actual and estimated portion size for Slavonian meat stew *"Čobanac"*

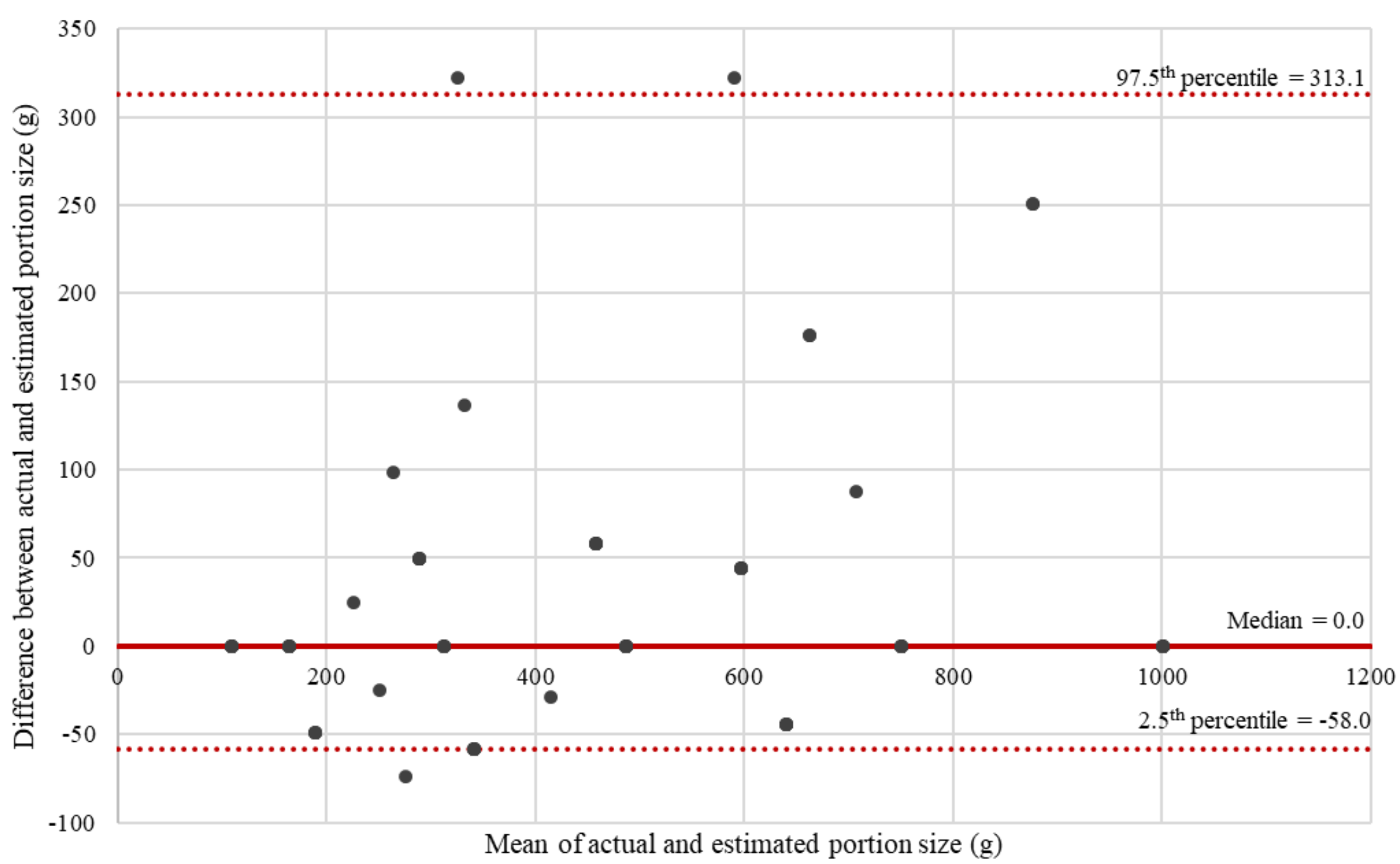

Supplementary Figure 20. Bland–Altman plots showing agreement between the actual and estimated portion size for cooked vegetables *"Sataraš"* with roasted meat

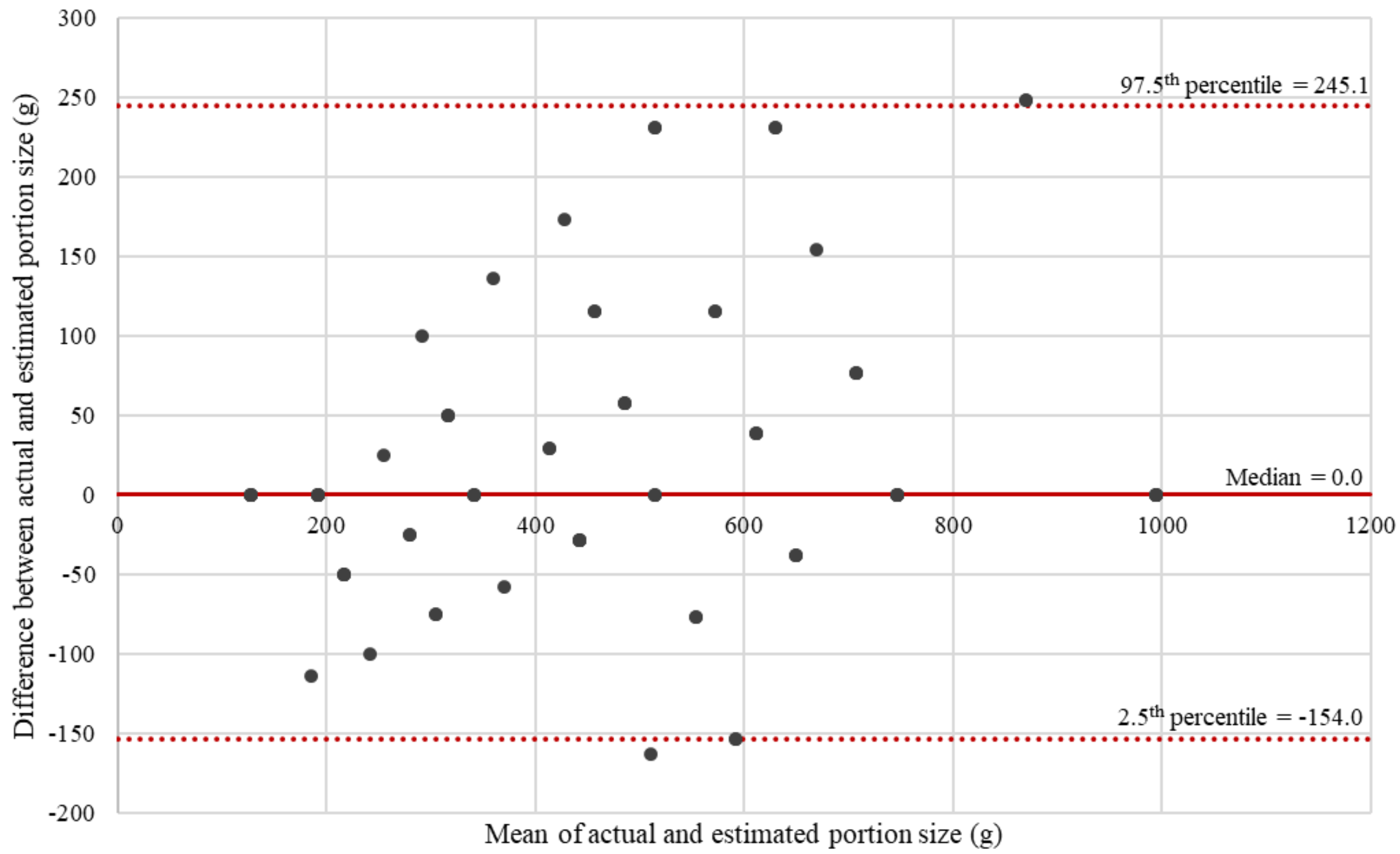

Supplementary Figure 21. Bland–Altman plots showing agreement between the actual and estimated portion size for roasted lamb with potatoes

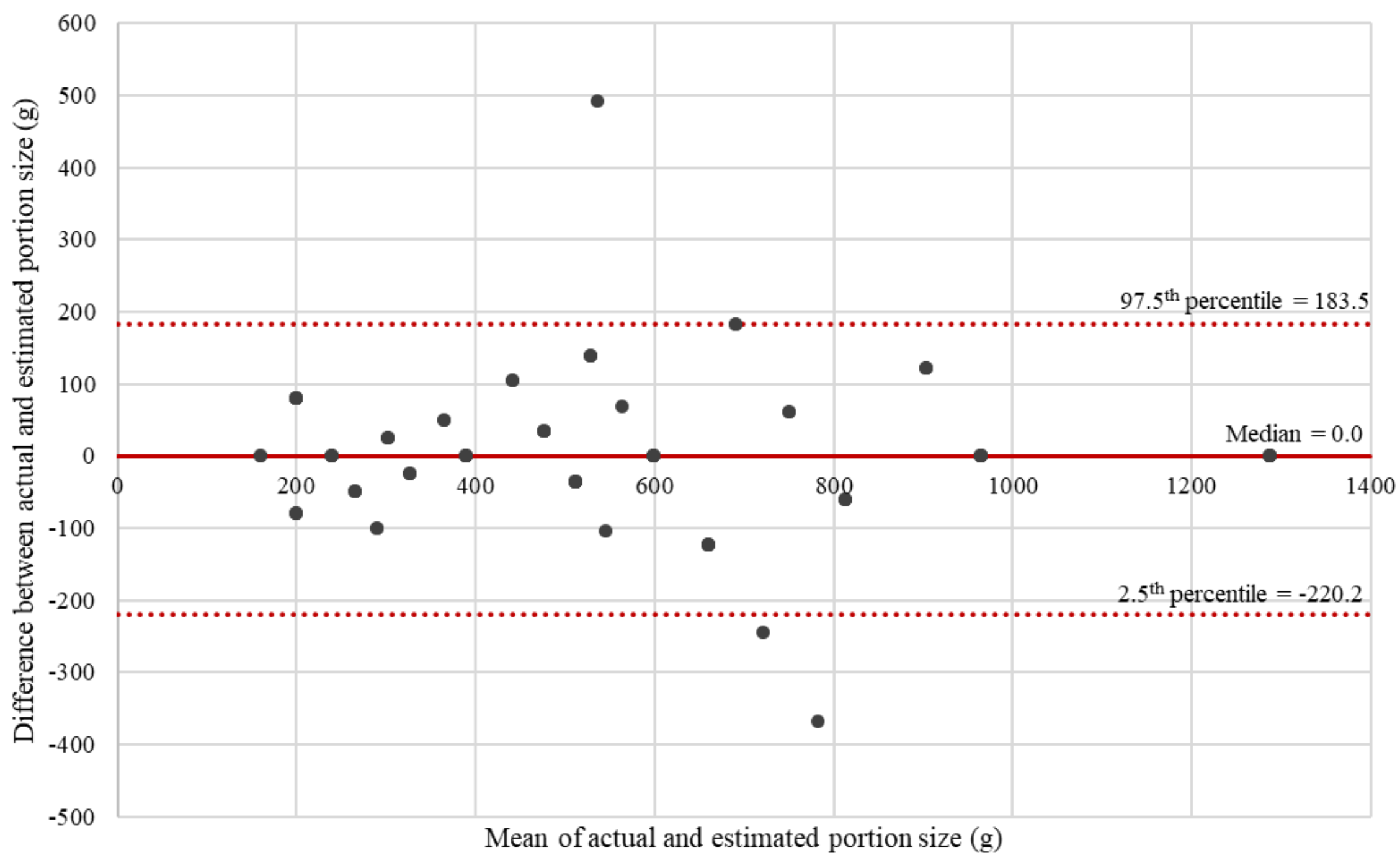

Supplementary Figure 22. Bland–Altman plots showing agreement between the actual and estimated portion size for stuffed pepper with mashed potatoes

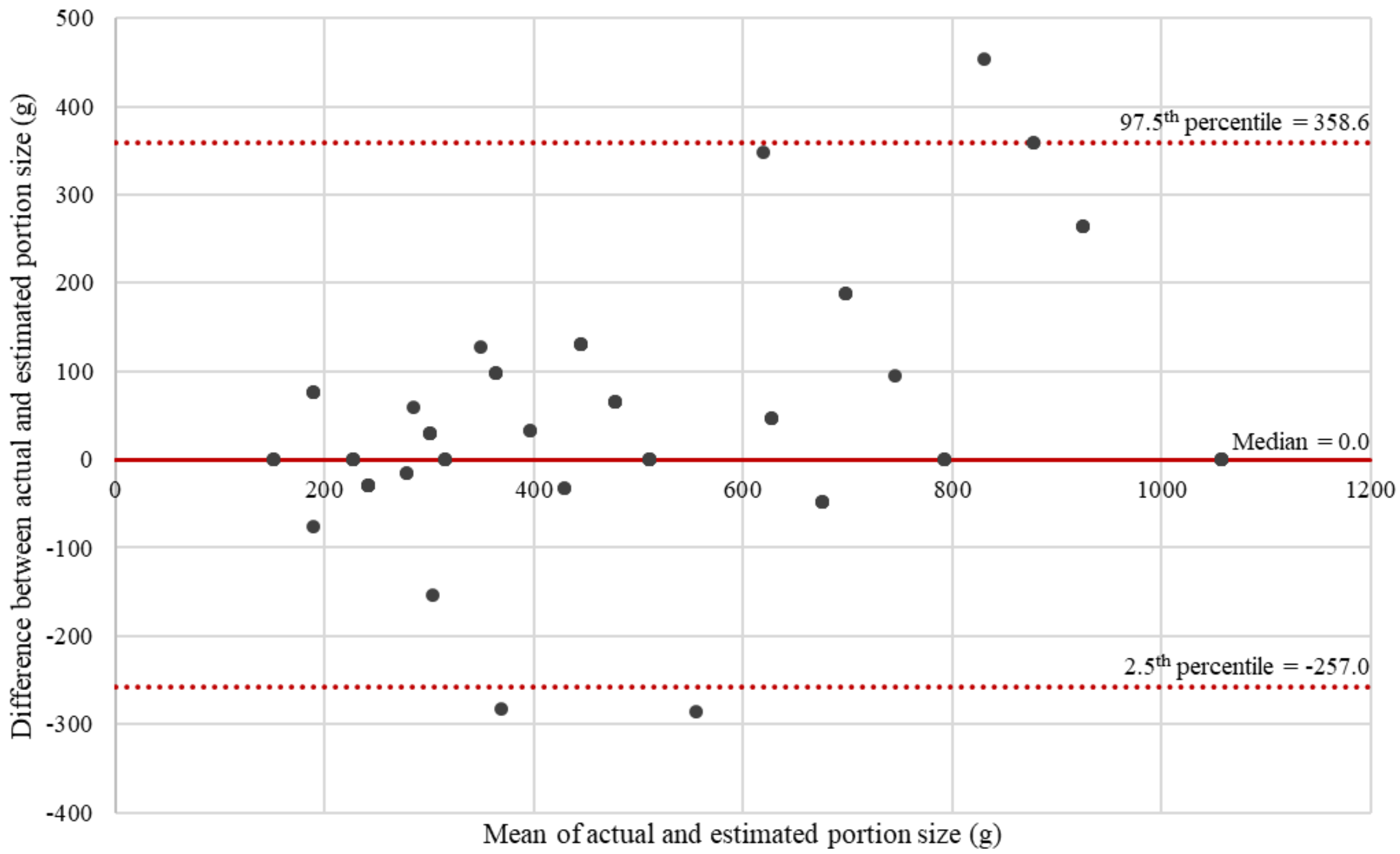

Supplementary Figure 23. Bland–Altman plots showing agreement between the actual and estimated portion size for stuffed cabbage rolls *"Sarma"* with mashed potatoes

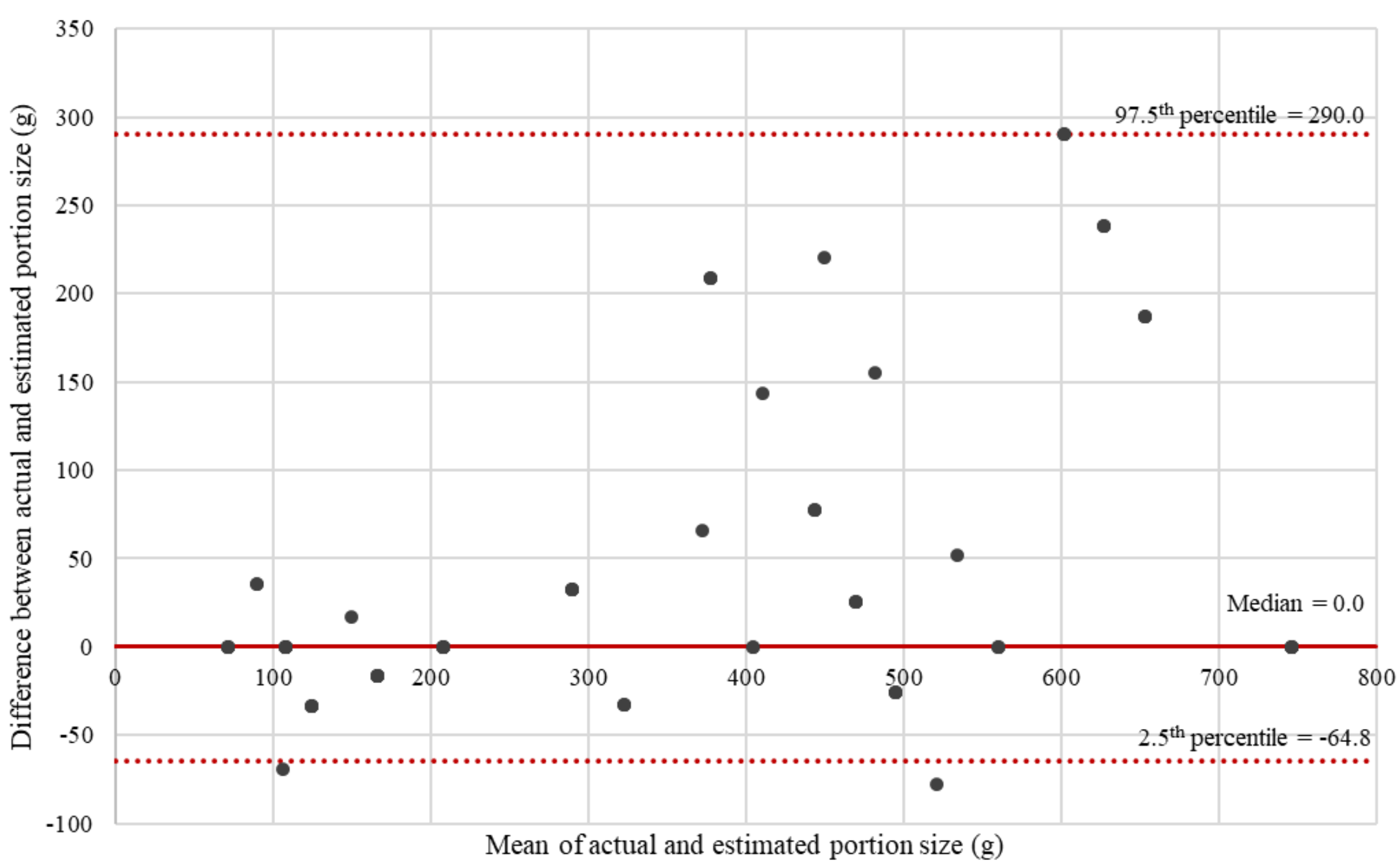

Supplementary Figure 24. Bland–Altman plots showing agreement between the actual and estimated portion size for kebabs *"Ćevapi"* in flatbread *"Lepinja"*

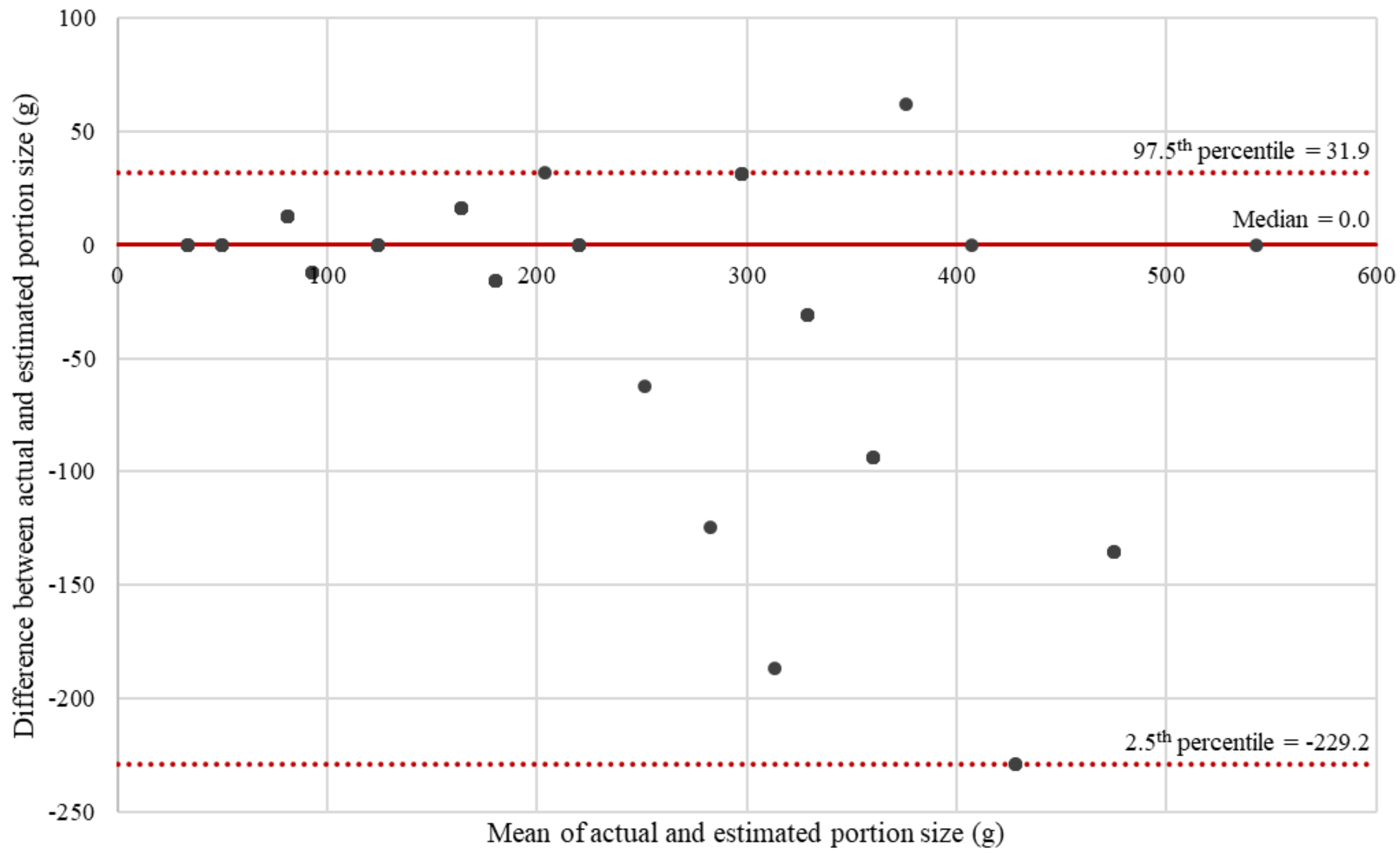

Supplementary Figure 25. Bland–Altman plots showing agreement between the actual and estimated portion size for kebabs *"Ćevapi"*

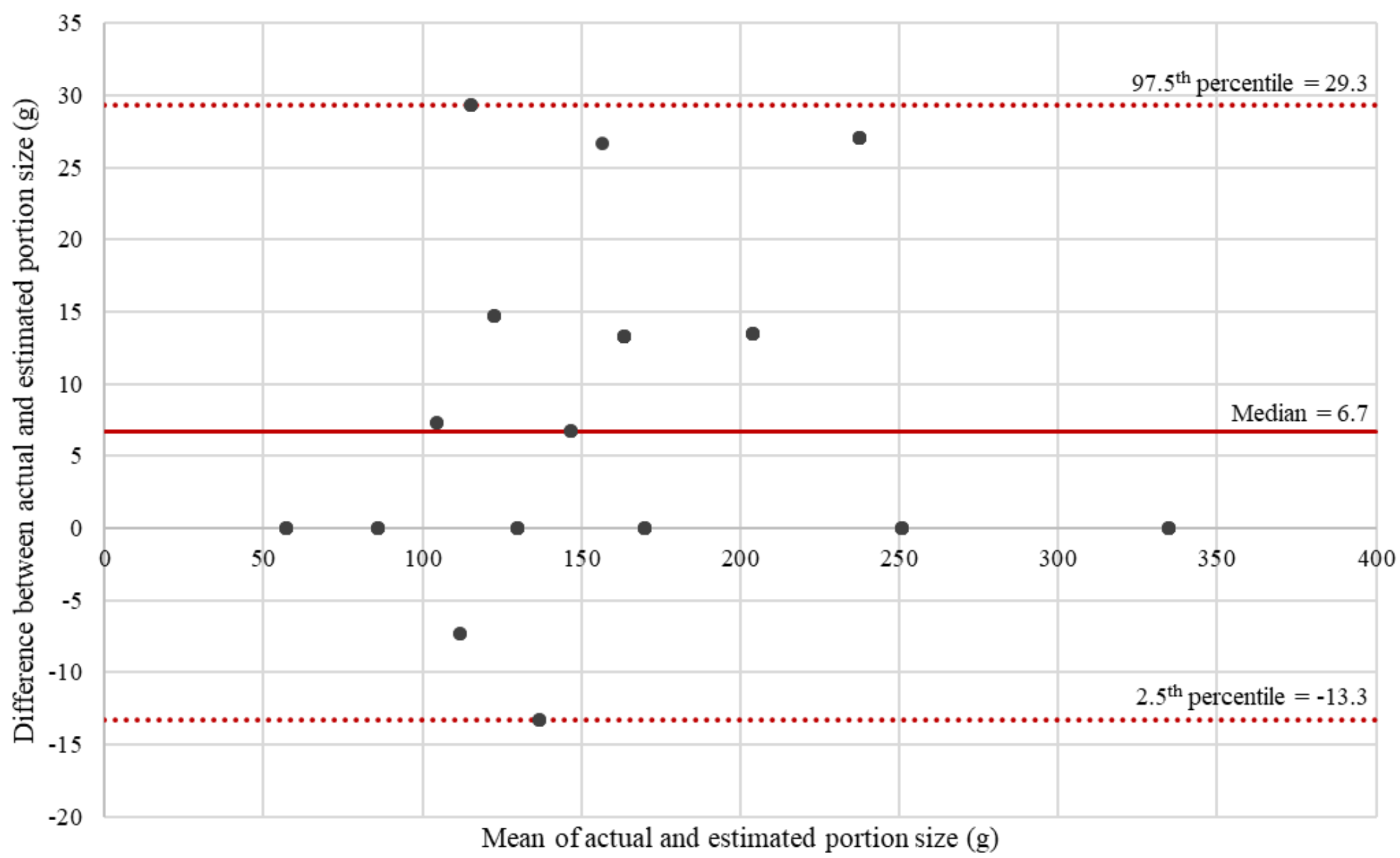

Supplementary Figure 26. Bland–Altman plots showing agreement between the actual and estimated portion size for walnut roll

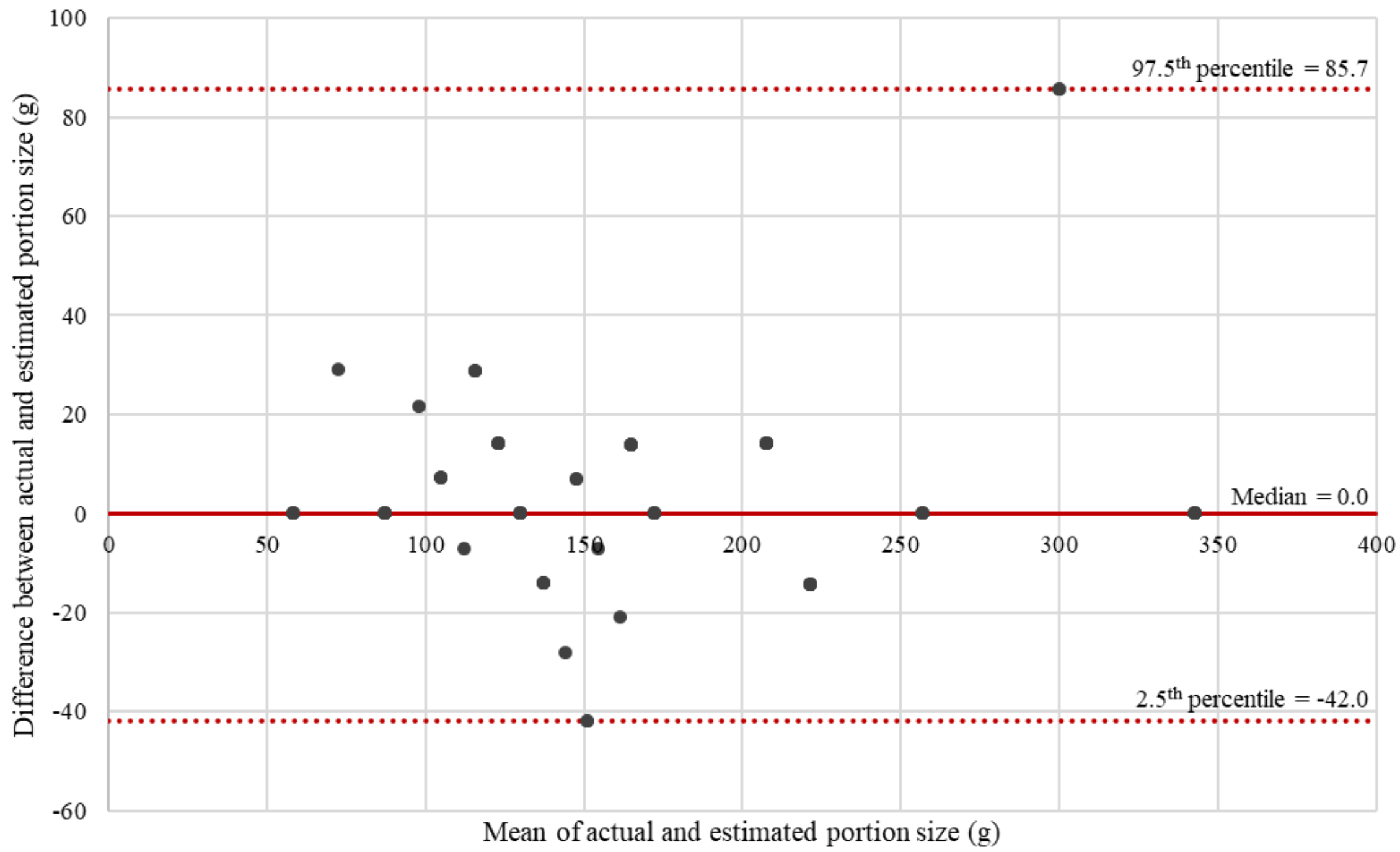

Supplementary Figure 27. Bland–Altman plots showing agreement between the actual and estimated portion size for poppy seed roll

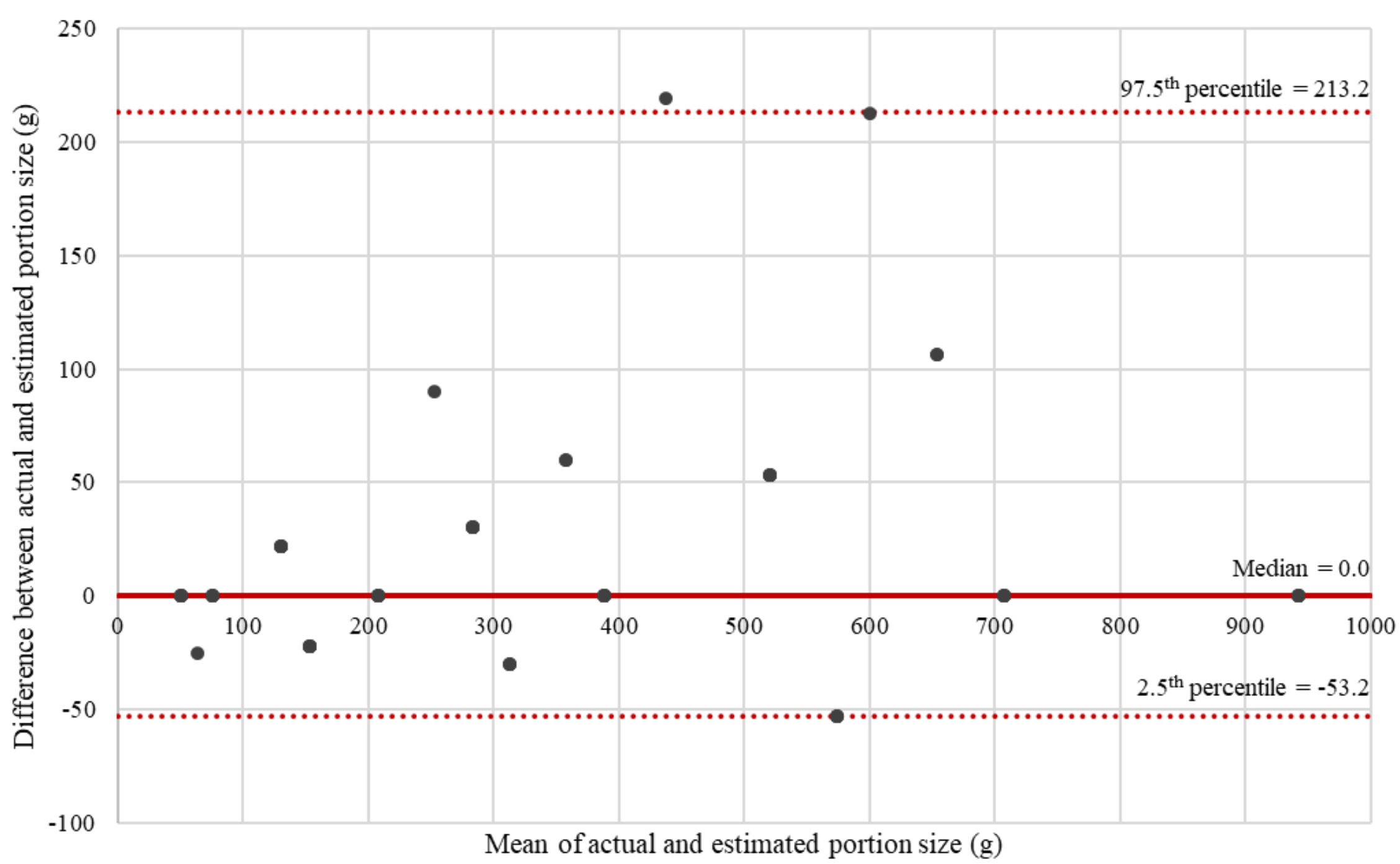

Supplementary Figure 28. Bland–Altman plots showing agreement between the actual and estimated portion size for dumplings stuffed with plum

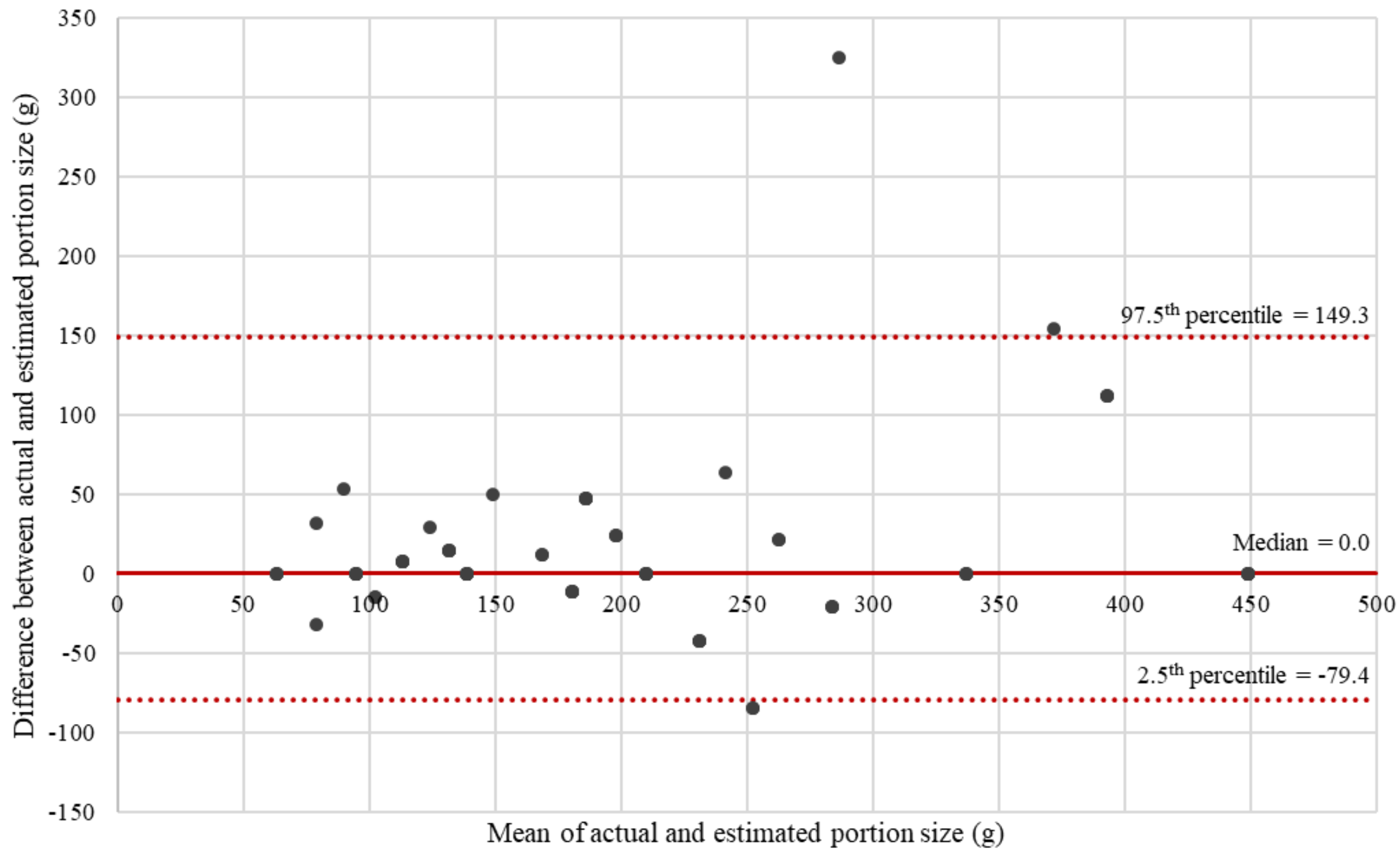

Supplementary Figure 29. Bland–Altman plots showing agreement between the actual and estimated portion size for custard slice *"Kremšnita"*

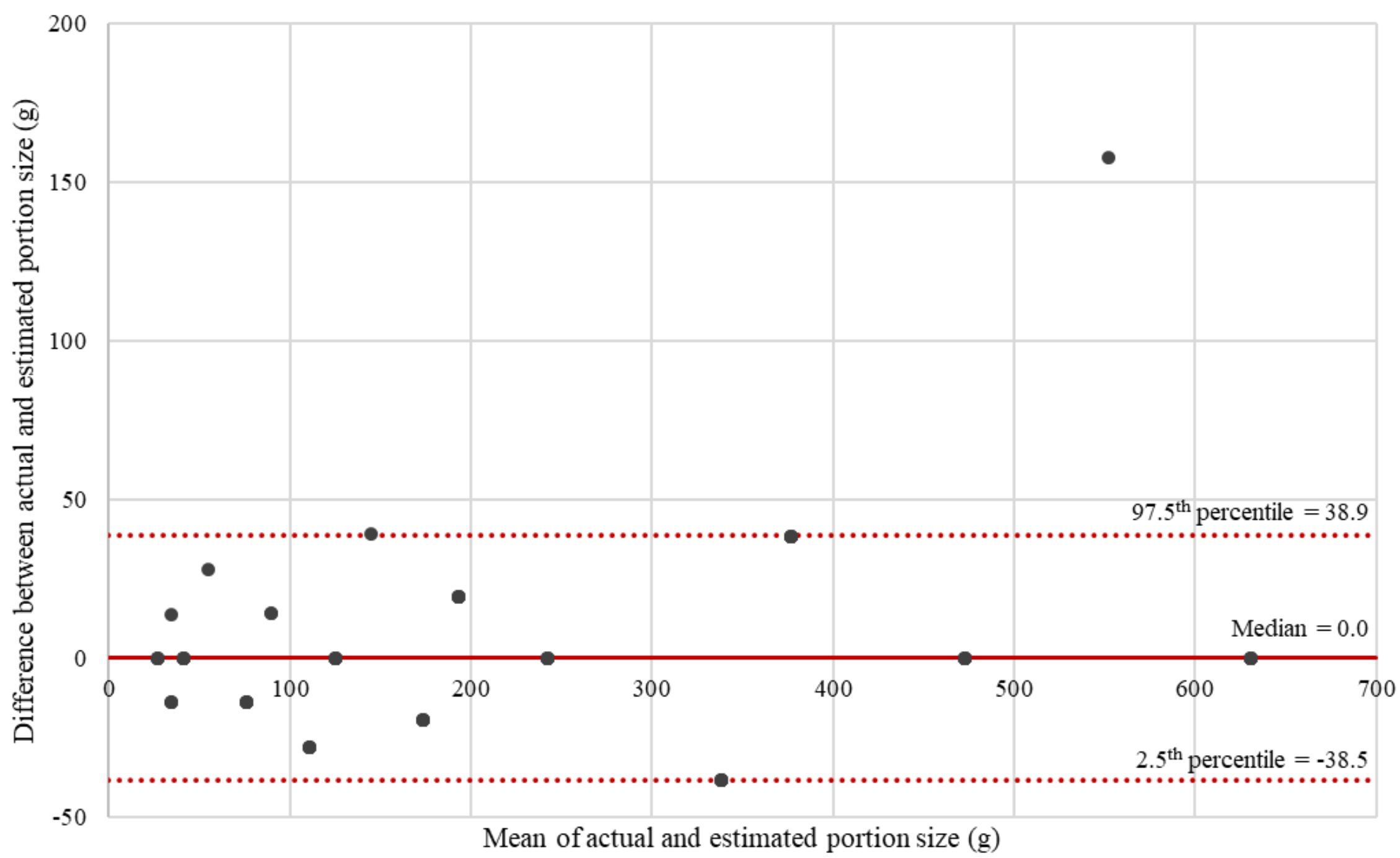

Supplementary Figure 30. Bland–Altman plots showing agreement between the actual and estimated portion size for pancakes
